# Evaluation of musculoskeletal models, scaling methods, and performance criteria for estimating muscle excitations and fiber lengths across walking speeds

**DOI:** 10.3389/fbioe.2022.1002731

**Published:** 2022-10-06

**Authors:** Israel Luis, Maarten Afschrift, Friedl De Groote, Elena M. Gutierrez-Farewik

**Affiliations:** ^1^ KTH MoveAbility Lab, Department of Engineering Mechanics, KTH Royal Institute of Technology, Stockholm, Sweden; ^2^ Department of Movement Sciences, KU Leuven, Leuven, Belgium; ^3^ Department of Women’s and Children’s Health, Karolinska Institutet, Stockholm, Sweden

**Keywords:** musculoskeletal modeling, optimal control, muscle-tendon parameter, performance criteria, EMG, fiber length

## Abstract

Muscle-driven simulations have been widely adopted to study muscle-tendon behavior; several generic musculoskeletal models have been developed, and their biofidelity improved based on available experimental data and computational feasibility. It is, however, not clear which, if any, of these models accurately estimate muscle-tendon dynamics over a range of walking speeds. In addition, the interaction between model selection, performance criteria to solve muscle redundancy, and approaches for scaling muscle-tendon properties remain unclear. This study aims to compare estimated muscle excitations and muscle fiber lengths, qualitatively and quantitatively, from several model combinations to experimental observations. We tested three generic models proposed by Hamner et al., Rajagopal et al., and Lai-Arnold et al. in combination with performance criteria based on minimization of muscle effort to the power of 2, 3, 5, and 10, and four approaches to scale the muscle-tendon unit properties of maximum isometric force, optimal fiber length, and tendon slack length. We collected motion analysis and electromyography data in eight able-bodied subjects walking at seven speeds and compared agreement between estimated/modelled muscle excitations and observed muscle excitations from electromyography and computed normalized fiber lengths to values reported in the literature. We found that best agreement in on/off timing in vastus lateralis, vastus medialis, tibialis anterior, gastrocnemius lateralis, gastrocnemius medialis, and soleus was estimated with minimum squared muscle effort than to higher exponents, regardless of model and scaling approach. Also, minimum squared or cubed muscle effort with only a subset of muscle-tendon unit scaling approaches produced the best time-series agreement and best estimates of the increment of muscle excitation magnitude across walking speeds. There were discrepancies in estimated fiber lengths and muscle excitations among the models, with the largest discrepancy in the Hamner et al. model. The model proposed by Lai-Arnold et al. best estimated muscle excitation estimates overall, but failed to estimate realistic muscle fiber lengths, which were better estimated with the model proposed by Rajagopal et al. No single model combination estimated the most accurate muscle excitations for all muscles; commonly observed disagreements include onset delay, underestimated co-activation, and failure to estimate muscle excitation increments across walking speeds.

## Introduction

Neuromusculoskeletal modeling enables the study of individual muscle-tendon unit (MTU) behavior, joint kinematics, dynamics, and neuromuscular control strategies by non-invasive means. Simulation studies have provided insights into the MTU mechanics in able-bodied individuals (Heintz and Gutierrez-Farewik, 2007; Hamner et al., 2010; Arnold et al., 2013; Swinnen et al., 2019; Delabastita et al., 2020), and in individuals with disabilities in unassisted motions (Krogt et al., 2016) and with the support of assistive devices (Lotti et al., 2020). A realistic representation of MTU behavior depends on adequately modeling skeletal anatomy and muscle architecture and decoding neuromuscular control. The most challenging aspect is to perform *in vivo* measurements, i.e., muscle and ligaments properties (Charles et al., 2019a), anatomical degrees of freedom (Zandbergen et al., 2020), or muscle dynamics, and to validate them with experimental observations (Hicks et al., 2015). As such, simplifications of the neuromusculoskeletal system are necessary. Consequently, it is critical to review and refine these methods to gain confidence in their use.

Neuromusculoskeletal modeling integrates musculoskeletal modeling, which describes the mechanics of the human biological system, and neuromuscular control, which synthesizes the principles for controlling such a system. Musculoskeletal models incorporate a description of the bone geometry, degrees of freedom, and body-segment physical properties: mass, inertia, and center of mass, and muscle geometry, architecture, and dynamics. Muscle dynamics is subdivided into activation and contraction dynamics (Zajac, 1989). Activation dynamics describes the relationship between the motor unit discharges from the nerve to the muscle, muscle excitation, and the concentration of calcium ions within the intracellular space, muscle activation. Contraction dynamics describes the force-generating capacity of the muscle and the behavior of connective tissue: tendon and aponeurosis and is commonly represented by a Hill-type model. This model includes parameters such as maximum isometric force (MIF), maximum contraction velocity (MCV), optimal fiber length (OFL), pennation angle (PA) at OFL, and tendon slack length (TSL). Muscles’ contractions are mainly responsible for the development of skeletal motion. However, their coordination pattern is not trivial as human movement is highly redundant; there are more muscles than degrees of freedom. To address this redundancy in neuromuscular control, it is typically assumed that human movement control is governed through optimizing some performance criterion. Therefore, muscle controls are estimated by solving an optimization problem. Different performance criteria, e.g., minimal muscle activation, joint force, metabolic demand, etc., have been proposed to solve for individual muscle forces (Heintz and Gutierrez-Farewik, 2007). The combination of musculoskeletal models and optimal control provides a framework for investigating MTU behavior underlying an observed movement pattern.

A series of generic musculoskeletal models have been proposed in previous decades, but a comprehensive comparison of how model choice influences the estimation of underlying muscle excitations involved in movement is lacking. Musculoskeletal models have progressively increased their physiological fidelity, initially developed from cadaveric databases, and currently integrating *in vivo* observation of MTU characteristics through biomedical imaging and computational optimization techniques. Several generic musculoskeletal models have been revised, updated, and implemented in biomechanical modeling and simulation software, such as OpenSim. Frequently used models include those by Delp et al. (Delp et al., 1990), Hamner et al. (Hamner et al., 2010), Arnold et al. (Arnold et al., 2010), Rajagopal et al. (Rajagopal et al., 2016), and Lai et al. (Lai et al., 2018). In pioneering work, Delp et al. (Delp et al., 1990) formulated a lower limb model with MTU parameter values mainly obtained from five cadavers and TSL estimation from the model itself. Hamner et al. (Hamner et al., 2010) incorporated torso and arm segments in the Delp et al. model to study muscle contributions during running. Arnold et al. (Arnold et al., 2010) further developed the model by updating bone geometry (Arnold et al., 2000) and by implementing muscle architecture from a more comprehensive dataset from 21 cadavers (Ward et al., 2009). This model allowed a detailed description of the operating range of multiple muscles (Arnold et al., 2013), for instance, the soleus, which was aligned with experimental findings (Rubenson et al., 2012). Rajagopal et al. (Rajagopal et al., 2016) further updated the model of Arnold et al. by incorporating the relationship between individual muscle volume and leg volume in able-bodied young adults (Handsfield et al., 2014) and included torso and arm segments. Rajagopal et al. reported that this model overestimated fiber length change and passive fiber force due to the limitation of modeling the MTU with only a one-dimensional path, and that the excessive passive forces led to anomalous compensatory muscle excitations at the knee (Rajagopal et al., 2016) and hip extensors (Lai et al., 2018). Lai et al. refined the Rajagopal model by increasing knee range of motion and updating the tibiofemoral kinematics, as well as attachment points, wrapping surfaces, and TSL and OFL of gastrocnemius lateralis, gastrocnemius medialis, gluteus maximus, rectus femoris, semimembranosus, soleus, vastus intermedius, vastus medialis, and vastus lateralis. Lai et al. reported that their model estimated lower muscle co-activation and muscle excitations that overall agreed better with recorded EMGs in walking, running, and pedalling (Lai et al., 2018) than the model by Rajagopal et al. This succession of models has incorporated more information about MTU geometry and parameters that are likely to estimate with increasing accuracy MTU time-dependent behavior, such as muscle excitation. However, validation has only been performed in a few physical activities. To the best of our knowledge, a quantitative evaluation and comparison of their biofidelity over a range of walking speeds, compared with experimental data, has not been performed.

A generic musculoskeletal model must be scaled to a subject’s anthropometry to be suitable for analyzing muscle behavior during measured movement patterns, though how to scale MTU parameters is not fully known. In OpenSim, scaling is performed based on a calibration trial, wherein dimensions of model segments are modified to fit the subject’s anthropometry. Muscle-tendon attachment points are scaled linearly, proportionally to generic and scaled model segment lengths, as are OFL and TSL, by maintaining the same OFL/TSL ratio. However, this procedure has questionable validity, as OFL has been reported to not correlate linearly to leg length (Charles et al., 2020). In addition, MIF is generally not scaled, which is also questionable as muscle volume (and indirectly physiological cross-sectional area) has been reported to vary with height and weight (Handsfield et al., 2014; Charles et al., 2019b), age, gender, among other factors (Thelen et al., 2003). Computational methods have been proposed to scale MTU parameters (Winby et al., 2008; Krogt et al., 2016; Modenese et al., 2016). Modenese et al. implemented an algorithm to estimate OFL and TSL based on mapping a muscle’s operating range of a generic model onto a scaled model (Modenese et al., 2016). Rajagopal et al. scaled the MIF based on individual muscle volume, which was in turn estimated based on a regression equation from a study using magnetic resonance imaging (Rajagopal et al., 2016), and van der Krogt et al. scaled MIF by taking into account the generic and scaled models’ mass ratio (Krogt et al., 2016). Despite the introduction of numerous scaling methods, studies that evaluate their effect in the estimation of the muscle-tendon dynamics are scarce. Recently Charles et al. reported a better estimation of joint torque during maximum voluntary contraction trials in a model that scaled MIF as per van der Krogt et al. and OFL/TSL as per Modenese et al., compared to generic MTU parameters (Charles et al., 2020). This finding provided evidence that supports MTU parameter scaling. However, it is unknown whether scaling methods can improve estimation of MTU mechanics in different musculoskeletal models.

Simulation studies examining performance criteria are also numerous. Ackermann and van den Bogert simulated motion pattern and foot-ground contact forces with a two-dimensional musculoskeletal model walking at 1.1 m/s and solved the redundancy using performance criteria of minimum sum of muscle activations to the powers of 1, 2, 3, and 10, and with or without scaling muscle volume, with an aim to identify which combination provide the best estimation of muscle excitation, joint kinematics, and ground reaction force (Ackermann and van den Bogert, 2010). The best agreement was obtained using minimum sum of muscle activation to the power of 10. Zargham et al. investigated the effect of multiple performance criteria on estimated muscle excitation in simulation with prescribed kinematics and dynamics obtained from subjects with instrumented knee prostheses at a self-selected speed (1 m/s) (Zargham et al., 2019). They found that performance criteria of minimum sum of muscle activations to a power of 3 or 4, with and without scaled muscle volumes, resulted in better agreement with experimental EMGs than other criteria such as minimizing muscle force or contact force. In addition, both studies were only performed at one walking speed, so it is unclear whereas performance criteria based on minimal muscle effort allow to estimate muscle activation trends across walking speed and which power better performs.

In summary, although multiple generic musculoskeletal have been developed, they have not been quantitatively examined to determine which most accurately estimates underlying muscle excitation in observed movements. It is also unclear how muscle model parameter scaling, and different performance criteria based on muscle effort influence MTU mechanics estimations. As such, the goals of this study were to compute muscle excitations with three different generic open-source models, using four different performance criteria based on muscle effort, and four different methods to scale MIF, OFL, and TSL, of walking at 7 different speeds, and to qualitatively and quantitively evaluate which combination(s) of model, scaling method, and performance criterion yields muscle excitations that agree best with experimentally observed EMGs in eight muscles. Another goal was to evaluate indicators of validity of prescribed musculoskeletal simulations by studying whether MTU actuators were capable of reproducing the inverse dynamic joint torques (Hicks et al., 2015). Subsequent goals were to compare estimated fiber lengths of vastus lateralis, tibialis anterior, gastrocnemius medialis and lateralis, and soleus with those reported in literature.

## Materials and methods

### Musculoskeletal models, MTU-scaled parameters, and performance criteria

Three generic models available on the SimTK website were examined, referred to as Hamner (Hamner et al., 2010), Rajagopal (Rajagopal et al., 2016), and Lai-Arnold (Lai et al., 2018) models. Each model was anisotropically scaled: muscle attachments, skeletal geometry, and segment inertial properties were scaled to anthropometric dimensions obtained from a static calibration trial from 3D motion capture. Model segments were scaled using the marker positions based on the Conventional Gait Model with the extended foot model (CGM 2.4).

MTU parameters of the Hill-type muscle models were scaled in 4 different ways. OFL and TSL were scaled in two ways - linearly by the ratio between MTU lengths in the scaled and generic model (anatomic position, OpenSim’s Scale Tool) or non-linearly to preserve the operating range of the muscles (Modenese et al. (Modenese et al., 2016)). For each of these, MIF was either unscaled (generic MIF) or scaled based on muscle volumes derived from a regression equation that relates MRI-based muscle volumes to the subject’s height and weight (Handsfield et al., 2014). Thus, for each individual, each of the 3 models was scaled in 4 scaling variants:

S1: Linear scaling of OFL and TSL, generic MIF.

S2: Non-linear scaling of OFL and TSL, generic MIF.

S3: Linear scaling of OFL and TSL, scaled MIF.

S4: Non-linear scaling of OFL and TSL, scaled MIF.

Scaled MIFs were computed based on the assumption that MIF is proportional to the physiological cross-sectional area (PCSA) and specific tension, as shown in Eq. 1

$$F_{Mi}^{0}=A_{i}\sigma =\frac{V_{M_{i}}V_{T}}{l_{Mi}^{0}}\sigma$$ (1)

Where $F_{Mi}^{0}$ is the maximum isometric force, $A_{i}$ is the physiological cross-sectional area, $l_{Mi}^{0}$ is the optimal fiber length, $V_{T}$ is the total leg volume, and $V_{M_{i}}$ is the percentage of muscle volume with respect to the total leg volume of the muscle i , both estimated from Handsfield et al. (Handsfield et al., 2014), and σ is the specific tension, which has a value of 60 N/cm^2^ for each muscle (Rajagopal et al., 2016). All models assume the same MCV (10 OFL/s), activation time constant (10 ms), and deactivation time constant (40 ms) for all muscles, and PA was as per the original models. In addition, the non-linear tendon stiffness of gastrocnemius medialis, gastrocnemius lateralis, and soleus were set to 15 (nondimensionalized), according to ultrasound studies (Swinnen et al., 2019; Delabastita et al., 2020) that describe the Achilles tendon as highly compliant. Similarly, the non-linear tendon stiffness of vastus lateralis, vastus medialis, vastus intermedius, and rectus femoris was set to 20 (nondimensionalized), according to findings that modelling the Patellar tendon as highly compliant agrees better with experimental observations (Chleboun et al., 2007; Bohm et al., 2018), including muscle fiber operating range (Son et al., 2018).

In addition, for each model and scaling variant, 4 performance criteria based on minimum muscle effort, defined as the sum of muscle activations and excitations to powers (*p*) of 2, 3, 5, and 10, were examined as shown in Eq. 2

$$J_{p}=\int_{t_{i}}^{t_{f}}(\overset{N}{\underset{i=1}{\sum }}\frac{\left(e_{i}^{p}\left(t\right)+a_{i}^{p}\left(t\right)\right)}{2})dt$$ (2)

Where $t_{i}$ is the initial time of the gait cycle, $t_{f}$ is the final time of the gait cycle, $e_{i}$ is muscle excitation of the muscle i , $a_{i}$ is muscle activation of the muscle i , and N the total number of muscles in one leg of each model. These performance criteria are referred to as J2, J3, J5, and J10

### Subjects

Eight able-bodied adults (5 males, 3 females, mean [SD] age: 37.8 [9.6] years, height: 1.76 [0.10] m, mass: 76.6 [14.4] kg) participated in this experiment as part of a more comprehensive study to characterize energetics in locomotion.

### Experimental setup and protocols

Subjects walked at different speeds based on the expected preferred walking speed (PWS), based on each subject’s gender, age, and height (Bohannon, 1997). The subjects walked on a treadmill at 55%, 70%, 85%, 100%, 115%, 130%, and 145% of the PWS in randomized order, and each subject’s cadence at each speed was recorded. Then, for overground walking along a walkway, each of the 7 walking speeds was estimated by matching the cadence of treadmill walking. Reflective marker positions (100 Hz) and ground reaction forces (1,000 Hz) were measured during overground walking using optical motion capture (Vicon V16, Oxford, United Kingdom) and strain gauge force platforms (AMTI, Watertown, MA, United States). Full-body marker placement was implemented based on the Conventional Gait Model with the extended foot model (CGM 2.4). EMGs from eight muscles in one leg were recorded (1,000 Hz): biceps femoris long head (BF), semitendinosus (ST), vastus lateralis (VL), vastus medialis (VM), tibialis anterior (TA), gastrocnemius lateralis (GL), gastrocnemius medialis (GM) and soleus (SO), using bipolar surface wireless electrodes (Myon aktos/Cometa systems, Milan, Italy). The selection of the leg side for each subject was randomized. Skin preparation and electrode placement followed Electromyography for the Non-Invasive Assessment of Muscles guidelines (SENIAM) (Hermens et al., 2000). Subjects were asked to perform functional tests, such as standing heel raise, standing toe raises, squat, knee flexion, and hip flexion/extension, to corroborate the placement of the EMGs.

### Data and statistical analysis

Generic models were scaled to each subject’s dimensions as described above. Marker trajectories and ground reaction forces during 21 gait cycles per subject (3 gait cycles at each of 7 walking speeds) were used in 3D inverse kinematics (IK) and inverse dynamics (ID) using OpenSim 4.1. IK tracking weights were selected to minimize the error between experimental markers and virtual markers placed on the model. For each subject, the same weights were used for each model, scaling variant and performance criteria. Subtalar and metatarsal joints were fixed at anatomically neutral positions. Muscle excitations and corresponding fiber lengths were estimated using a direct collocation dynamic optimization algorithm that incorporates activation and contraction dynamics (De Groote et al., 2016). IK and ID solutions were prescribed, and the objective function consisted of three terms: the first term refers to the muscle effort, as described above, the second term to the torque produced by reserve actuators, i.e., ideal actuators at each joint that served to generate the joint torques that the MTU actuators are not able to reproduce, and the third term, to the fiber velocity to improve the numerical computation. The mathematical expression of the objective function is shown in Eq. 3

$${J=w}_{e}J_{p}+w_{r}\int_{t_{i}}^{t_{f}}\left(\sum_{j=1}^{J}e_{Rj}^{2}\left(t\right)\right)dt+w_{v}\int_{t_{i}}^{t_{f}}\left(\overset{N}{\underset{i=1}{\sum }}v_{i}^{2}\left(t\right)\right)dt$$ (3)

Where $e_{Rj}$ is the reserve actuator of the joint j , $v_{i}$ is the muscle velocity of the muscle i , J the total number of joints in one leg of each model, and $w_{e}$ , $w_{r}$ , and $w_{v}$ are the weight of the terms related to muscle effort, reserve actuators, and fiber velocities, respectively. The value of $w_{e}$ is 1, the value of $w_{r}$ is 1,000, and $w_{v}$ is 0.01. As such, the use of the reserve actuators was highly penalized in the objective function, and the influence of the fiber velocity was relatively small. MTU parameters MIF, OFL, TSL, and tendon compliance, defined as OFL/TFL, as well as muscle excitations were computed for each gait cycle for each of the 3 musculoskeletal models, the 4 scaling variants (S1, S2, S3, and S4), and the 4 performance criteria (J2, J3, J5, and J10), which resulted in 48 estimates per gait cycle.

Recorded EMGs were processed using a fourth order zero-lag Butterworth band-pass filter (20–400 Hz), full-wave rectification, and a fourth order zero-lag Butterworth low-pass filter (6 Hz). Experimental muscle excitations were processed throughout the same 21 gait cycles per subject described above.

Scaled values of OFL, and TSL of each model and scaling variant were compared to experimental values reported by Ward et al. (Ward et al., 2009). Also, excessive reliance on reserve actuators to reproduce inverse dynamic joint torque was reported for each model combination.

Qualitative evaluation of estimated muscle excitation was performed by examining on/off timing agreement, defined as the period during which muscles are active, i.e., when EMGs or computed muscle excitations exceed a threshold, in this case 50% of the maximum value during each gait cycle.

Quantitative evaluation was performed by examining the agreement between excitation patterns and average excitation increments across walking speeds between EMGs and estimated muscle excitations. Time-series agreement was evaluated by computing correlation coefficients (r) and root-mean-squared error (RMSE) between normalized EMGs and estimated muscle excitations. Both EMGs and estimated excitations were normalized to the maximum value during that gait cycle. As such, we only evaluated correspondence of the pattern (Zargham et al., 2019) but not of the magnitude.

We also computed excitation/speed increment rate m as the rate at which the average muscle excitation increased with walking speed *via* a linear regression between the average excitation magnitude and walking speed. Thus, m was computed from average EMG values ( $m_{\exp}$ ) and from the simulations ( $m_{sim}$ ) for each model combination. Both observed and estimated muscle excitations were normalized to their average value observed during walking at PWS; thus $m_{\exp}$ ≈1 and $m_{sim}$ ≈1 at PWS. Increment rate disagreement $m_{d}$ between $m_{\exp}$ and $m_{sim}$ was evaluated as their difference, proportional to $m_{\exp}$ and as a percent, shown in Eq. 4

$$m_{d}=\left(\frac{m_{sim}-m_{exp}}{m_{exp}}\right)\cdot 100\left[\%\right]$$ (4)

Therefore, $m_{d}$ >0 indicates that the model overestimated excitation/speed increments, $m_{d}$ = 0 indicates that the model’s excitation/speed increments were identical to those from EMGs, and $m_{d}$ <0 indicates that the underestimated excitation/speed increments.

Finally, the fiber lengths were estimated for each model combination and compared to reported values; estimated normalized fiber lengths of VL, TA, and GL at PWS, as well as GM and SO at low, normal, and fast walking speeds, were compared to experimental values reported available in the literature. To facilitate the comparison, the normalized fiber length was divided into four categories based on muscle fiber length (L_M_) relative to optimal fiber length (OFL): steep ascending limb (L_M_/OFL< 0.75), shallow ascending limb (0.75 ≤L_M_/OFL < 0.95), plateau (0.95 ≤L_M_/OFL < 1.05), and descending limb (1.05< L_M_/OFL) (Arnold and Delp, 2011).

## Results

We found that muscle parameters estimated with scaled OFL and TSL were similar to those reported by Ward et al. (Ward et al., 2009) in most muscles and in all models. Scaling MIF had a larger effect on estimated muscle parameters in the Hamner model than in the other models (Table 1). Estimated OFLs with OFL and TSL linearly (S1) and non-linearly (S2) scaled in the Rajagopal model were within the expected values reported by Ward et al. (Ward et al., 2009), whereas VL, TA, and GM in the Hamner model, and GL in the Lai-Arnold model were not (Supplementary Table S1). OFL tended to be smaller with non-linearly scaled OFL and TSL in TA, GL, GM, and SO, but larger in BF and ST than linearly scaled OFL and TSL in all models. MTU compliance tended to be higher with non-linearly scaled in TA, GL, GM, and SO and lower in BF and ST than with linearly scaled OFL and TSL in all models. The Rajagopal and Lai-Arnold models estimated higher MIF than the Hamner model, with or without scaled MIF, for most muscles, except GL, VL, and VM.

**TABLE 1:** Average and SD of MIF [N], OFL [cm], TSL [cm], and MTU compliance, defined as TSL/OFL, among all subjects scaling variants: linear scaling of OFL and TSL and generic MIF (S1), non-linear scaling of OFL and TSL and generic MIF (S2), linear scaling of OFL and TSL and scaled MIF (S3), and non-linear scaling of OFL and TSL and scaled MIF (S4), in Hamner (H), Rajagopal (R), and Lai-Arnold (L) models in 8 muscles.

		S1				S2				S3				S4			
		MIF	OFL	TSL	TSL/OFL	MIF	OFL	TSL	TSL/OFL	MIF	OFL	TSL	TSL/OFL	MIF	OFL	TSL	TSL/OFL
BF	H	896.0 [0.0]	10.6 [0.6]	31.7 [1.9]	3.0 [0.0]	896.0 [0.0]	11.6 [0.8]	30.6 [1.8]	2.6 [0.2]	1,207.7 [212.3]	10.6 [0.6]	31.7 [1.9]	3.0 [0.0]	1,096.9 [146.6]	11.6 [0.8]	30.6 [1.8]	2.6 [0.2]
	R	1,313.2 [0.0]	9.4 [0.4]	31.4 [1.4]	3.3 [0.0]	1,313.2 [0.0]	10.0 [0.7]	31.0 [1.3]	3.1 [0.2]	1,357.6 [251.3]	9.4 [0.4]	31.4 [1.4]	3.3 [0.0]	1,274.5 [174.4]	10.0 [0.7]	31.0 [1.3]	3.1 [0.2]
	L	1,313.2 [0.0]	9.4 [0.4]	32.2 [1.4]	3.4 [0.0]	1,313.2 [0.0]	10.0 [0.7]	31.7 [1.3]	3.2 [0.2]	1,356.4 [250.1]	9.4 [0.4]	32.2 [1.4]	3.4 [0.0]	1,273.7 [173.6]	10.0 [0.7]	31.7 [1.3]	3.2 [0.2]
ST	H	410.0 [0.0]	19.7 [1.2]	25.0 [1.5]	1.3 [0.0]	410.0 [0.0]	21.2 [1.6]	23.2 [1.5]	1.1 [0.1]	579.2 [101.1]	19.7 [1.2]	25.0 [1.5]	1.3 [0.0]	535.2 [71.2]	21.2 [1.6]	23.2 [1.5]	1.1 [0.1]
	R	591.3 [0.0]	18.8 [0.9]	24.1 [1.1]	1.3 [0.0]	591.3 [0.0]	19.8 [1.4]	23.1 [1.2]	1.2 [0.1]	607.4 [111.3]	18.8 [0.9]	24.1 [1.1]	1.3 [0.0]	574.7 [79.1]	19.8 [1.4]	23.1 [1.2]	1.2 [0.1]
	L	591.3 [0.0]	18.8 [0.9]	24.0 [1.1]	1.3 [0.0]	591.3 [0.0]	19.8 [1.4]	23.0 [1.2]	1.2 [0.1]	607.6 [111.3]	18.8 [0.9]	24.0 [1.1]	1.3 [0.0]	574.1 [78.4]	19.8 [1.4]	23.0 [1.2]	1.2 [0.1]
VL	H	1871.0 [0.0]	8.2 [0.4]	15.4 [0.8]	1.9 [0.0]	1871.0 [0.0]	8.1 [0.5]	15.5 [0.8]	1.9 [0.0]	6,217.2 [1,089.6]	8.2 [0.4]	15.4 [0.8]	1.9 [0.0]	6,319.3 [1,072.8]	8.1 [0.5]	15.5 [0.8]	1.9 [0.0]
	R	5,148.8 [0.0]	9.8 [0.4]	21.7 [0.9]	2.2 [0.0]	5,148.8 [0.0]	9.5 [0.3]	21.8 [1.1]	2.3 [0.1]	5,235.3 [923.8]	9.8 [0.4]	21.7 [0.9]	2.2 [0.0]	5,387.7 [1,034.2]	9.5 [0.3]	21.8 [1.1]	2.3 [0.1]
	L	5,148.8 [0.0]	11.5 [0.5]	21.7 [0.9]	1.9 [0.0]	5,148.8 [0.0]	11.6 [0.3]	21.6 [1.1]	1.8 [0.1]	4,446.5 [783.0]	11.5 [0.5]	21.7 [0.9]	1.9 [0.0]	4,392.6 [823.4]	11.6 [0.3]	21.6 [1.1]	1.8 [0.1]
VM	H	1,294.0 [0.0]	8.6 [0.5]	12.2 [0.6]	1.4 [0.0]	1,294.0 [0.0]	8.8 [0.5]	12.1 [0.7]	1.4 [0.0]	3,089.2 [540.7]	8.6 [0.5]	12.2 [0.6]	1.4 [0.0]	3,006.2 [514.5]	8.8 [0.5]	12.1 [0.7]	1.4 [0.0]
	R	2,747.8 [0.0]	9.5 [0.4]	19.5 [0.9]	2.1 [0.0]	2,747.8 [0.0]	9.4 [0.3]	19.6 [1.0]	2.1 [0.1]	2,808.4 [496.6]	9.5 [0.4]	19.5 [0.9]	2.1 [0.0]	2,835.1 [531.8]	9.4 [0.3]	19.6 [1.0]	2.1 [0.1]
	L	2,747.8 [0.0]	10.8 [0.5]	20.3 [0.9]	1.9 [0.0]	2,747.8 [0.0]	11.1 [0.3]	20.1 [1.0]	1.8 [0.0]	2,470.2 [436.0]	10.8 [0.5]	20.3 [0.9]	1.9 [0.0]	2,390.4 [437.8]	11.1 [0.3]	20.1 [1.0]	1.8 [0.0]
TA	H	905.0 [0.0]	10.1 [0.7]	23.1 [1.7]	2.3 [0.0]	905.0 [0.0]	10.0 [0.8]	23.2 [1.6]	2.3 [0.1]	824.5 [126.9]	10.1 [0.7]	23.1 [1.7]	2.3 [0.0]	839.6 [125.7]	10.0 [0.8]	23.2 [1.6]	2.3 [0.1]
	R	1,227.5 [0.0]	7.0 [0.5]	24.7 [1.7]	3.5 [0.0]	1,227.5 [0.0]	6.9 [0.5]	24.7 [1.6]	3.6 [0.1]	1,193.9 [189.4]	7.0 [0.5]	24.7 [1.7]	3.5 [0.0]	1,203.0 [181.2]	6.9 [0.5]	24.7 [1.6]	3.6 [0.1]
	L	1,227.5 [0.0]	7.0 [0.5]	24.6 [1.6]	3.5 [0.0]	1,227.5 [0.0]	6.9 [0.5]	24.7 [1.6]	3.6 [0.1]	1,195.4 [190.0]	7.0 [0.5]	24.6 [1.6]	3.5 [0.0]	1,204.6 [181.7]	6.9 [0.5]	24.7 [1.6]	3.6 [0.1]
GL	H	683.0 [0.0]	6.6 [0.5]	39.2 [2.9]	5.9 [0.0]	683.0 [0.0]	6.2 [0.5]	39.6 [3.1]	6.4 [0.4]	1,397.9 [212.7]	6.6 [0.5]	39.2 [2.9]	5.9 [0.0]	1,487.2 [241.9]	6.2 [0.5]	39.6 [3.1]	6.4 [0.4]
	R	1,575.1 [0.0]	6.0 [0.4]	38.3 [2.6]	6.4 [0.0]	1,575.1 [0.0]	5.9 [0.4]	38.4 [2.7]	6.5 [0.3]	1,541.7 [239.4]	6.0 [0.4]	38.3 [2.6]	6.4 [0.0]	1,564.4 [266.3]	5.9 [0.4]	38.4 [2.7]	6.5 [0.3]
	L	1,575.1 [0.0]	7.0 [0.5]	38.0 [2.6]	5.4 [0.0]	1,575.1 [0.0]	6.8 [0.5]	38.2 [2.7]	5.6 [0.3]	1,315.8 [204.7]	7.0 [0.5]	38.0 [2.6]	5.4 [0.0]	1360.1 [233.7]	6.8 [0.5]	38.2 [2.7]	5.6 [0.3]
GM	H	1,558.0 [0.0]	6.2 [0.5]	40.2 [3.0]	6.5 [0.0]	1,558.0 [0.0]	5.9 [0.5]	40.5 [3.1]	6.9 [0.4]	2,558.9 [389.7]	6.2 [0.5]	40.2 [3.0]	6.5 [0.0]	2,710.8 [440.7]	5.9 [0.5]	40.5 [3.1]	6.9 [0.4]
	R	3,115.5 [0.0]	5.2 [0.4]	40.6 [2.7]	7.8 [0.0]	3,115.5 [0.0]	5.1 [0.4]	40.7 [2.8]	7.9 [0.4]	3,047.9 [474.5]	5.2 [0.4]	40.6 [2.7]	7.8 [0.0]	3,097.4 [533.9]	5.1 [0.4]	40.7 [2.8]	7.9 [0.4]
	L	3,115.5 [0.0]	6.0 [0.4]	39.4 [2.6]	6.6 [0.0]	3,115.5 [0.0]	5.8 [0.4]	39.6 [2.7]	6.8 [0.4]	2,635.5 [409.9]	6.0 [0.4]	39.4 [2.6]	6.6 [0.0]	2,731.9 [479.6]	5.8 [0.4]	39.6 [2.7]	6.8 [0.4]
SO	H	3,549.0 [0.0]	5.2 [0.4]	25.8 [1.9]	5.0 [0.0]	3,549.0 [0.0]	4.9 [0.5]	26.0 [2.0]	5.3 [0.5]	5,262.4 [802.3]	5.2 [0.4]	25.8 [1.9]	5.0 [0.0]	5,537.9 [875.6]	4.9 [0.5]	26.0 [2.0]	5.3 [0.5]
	R	6,194.8 [0.0]	4.5 [0.3]	28.3 [2.0]	6.3 [0.0]	6,194.8 [0.0]	4.4 [0.4]	28.4 [2.0]	6.5 [0.5]	6,032.5 [935.0]	4.5 [0.3]	28.3 [2.0]	6.3 [0.0]	6,172.5 [1,047.2]	4.4 [0.4]	28.4 [2.0]	6.5 [0.5]
	L	6,194.8 [0.0]	4.5 [0.3]	28.8 [2.0]	6.4 [0.0]	6,194.8 [0.0]	4.4 [0.4]	28.8 [2.0]	6.6 [0.6]	6,041.8 [938.1]	4.5 [0.3]	28.8 [2.0]	6.4 [0.0]	6,192.7 [1,050.8]	4.4 [0.4]	28.8 [2.0]	6.6 [0.6]

As an indicator of simulation validity, the number of gait cycles that required excessive reserve actuators, defined as >2.5 N·m, were computed (Supplementary Table S2). This threshold represented approximately 5% of the average peak values of all the joint torques at the slowest walking speed. Acceptable reserve actuators were achieved in most muscles with most of the model combinations. The Hamner model required higher reserve actuators when MIF was not scaled (S1 and S2), compared to the Rajagopal and Lai-Arnold model using any scaling variant. Notably, higher reserve actuator magnitudes were found in the Hamner model at the ankle and hip joint in the sagittal plane, particularly at high walking speeds (not reported).

**FIGURE 1:**
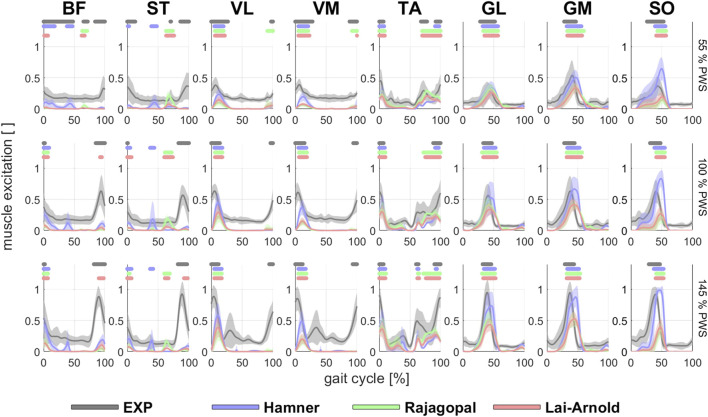
EMGs and estimated/modelled muscle excitations (average +/- 1 SD of all subjects) in 8 muscles with performance criterion J2 and scaling variant S1 at walking speeds of 55%, 100% and 145% PWS. EMGs were normalized to the maximum value at 145% PWS. Horizontal lines above each time series indicate on/off timing for EMG and each model, defined as >50% excitation.

**FIGURE 2:**
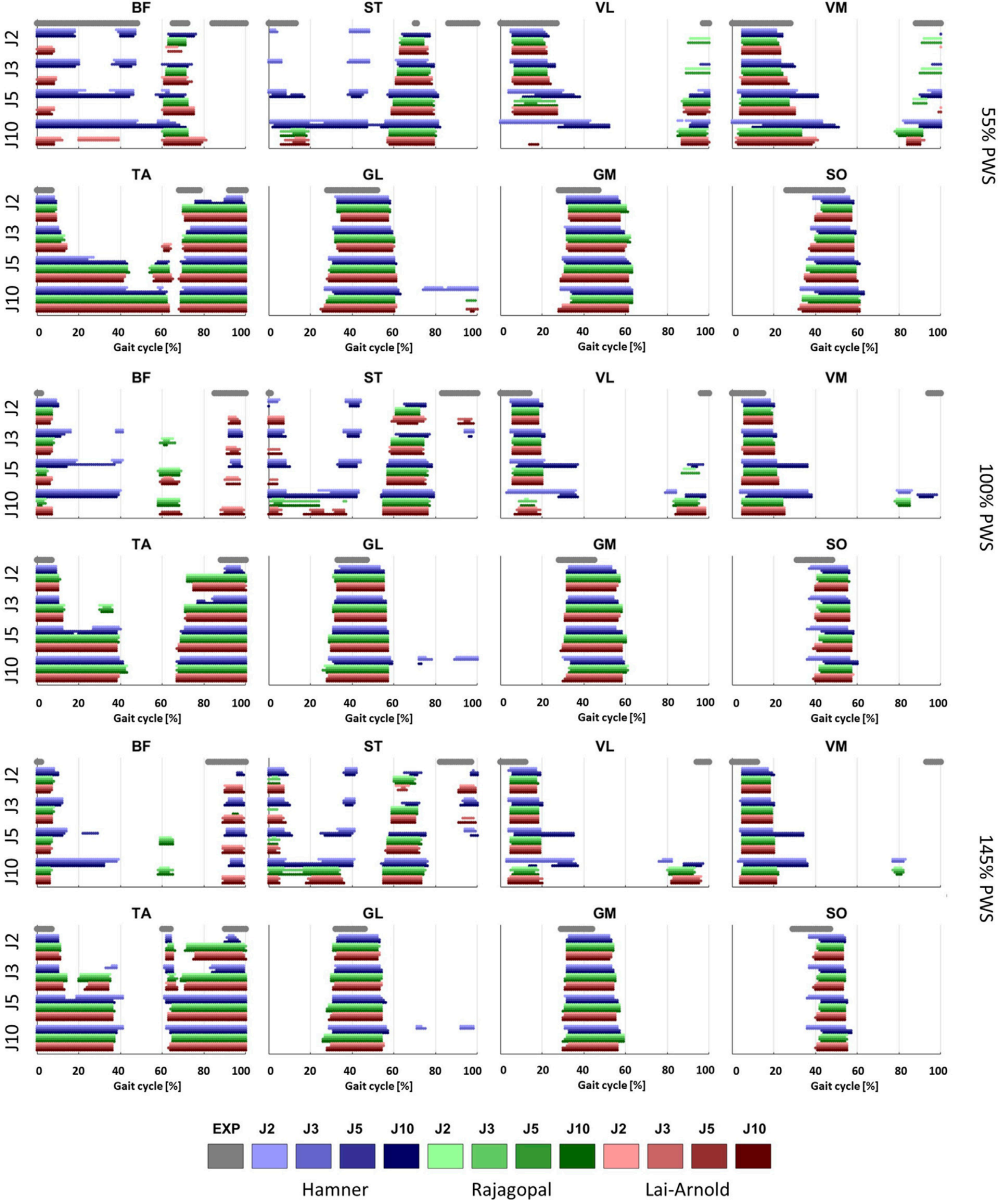
On/off timing of EMGs, averaged over all subjects, and estimated muscle excitations of 8 muscles at 3 walking speeds, with performance criteria based on minimization of muscle effort to the power of 2 (J2), 3 (J3), 5 (J5) and 10 (J10), and scaling variants with linear scaling of OFL and TSL and generic MIF (S1), non-linear scaling of OFL and TSL and generic MIF (S2), linear scaling of OFL and TSL and scaled MIF (S3), and non-linear scaling of OFL and TSL and scaled MIF (S4).

Estimated on/off timing in most muscles was similar for all model combinations. Estimated muscle excitations from all model combinations presented some similar discrepancies to EMGs, including lower co-activation and an excitation delay. Estimated on/off timing agreed well with EMGs for most models and across walking speeds in ankle plantar- and dorsiflexors TA, GL, GM, and SO, and to a lesser degree, in knee extensors VL and VM, with performance criterion J2 and with any scaling variant (Figures 1, 2). Estimated on/off timing in GL, GM, and SO for all models agreed well with EMGs and did not substantially change among speeds, nor were they substantially influenced by performance criterion or scaling variant. Estimated on/off timing in VL, VM, and TA agreed least with EMGs with J10 compared to other performance criteria (Supplementary Figure S1), particularly in the Hamner model (Figure 2). All model combinations failed to capture VL and VM excitation in late swing at high speeds. Estimated ST excitation with all model combinations agreed poorly with EMGs across speeds, wherein on/off timing varied throughout the gait cycle among models, particularly in the Hamner model. Similar disagreements with EMGs can be observed for a few muscles in all models. For instance, all model combinations estimated very low or no excitation within certain periods of the gait cycle, whereas average EMG values never reached zero excitation. Thus, all model combinations estimated less co-activation than were observed in EMGs. In addition, for all model combinations, VL, VM, GL, GM, and SO estimates closely resembled EMG patterns, but with a delayed onset across all speeds.

**FIGURE 3:**
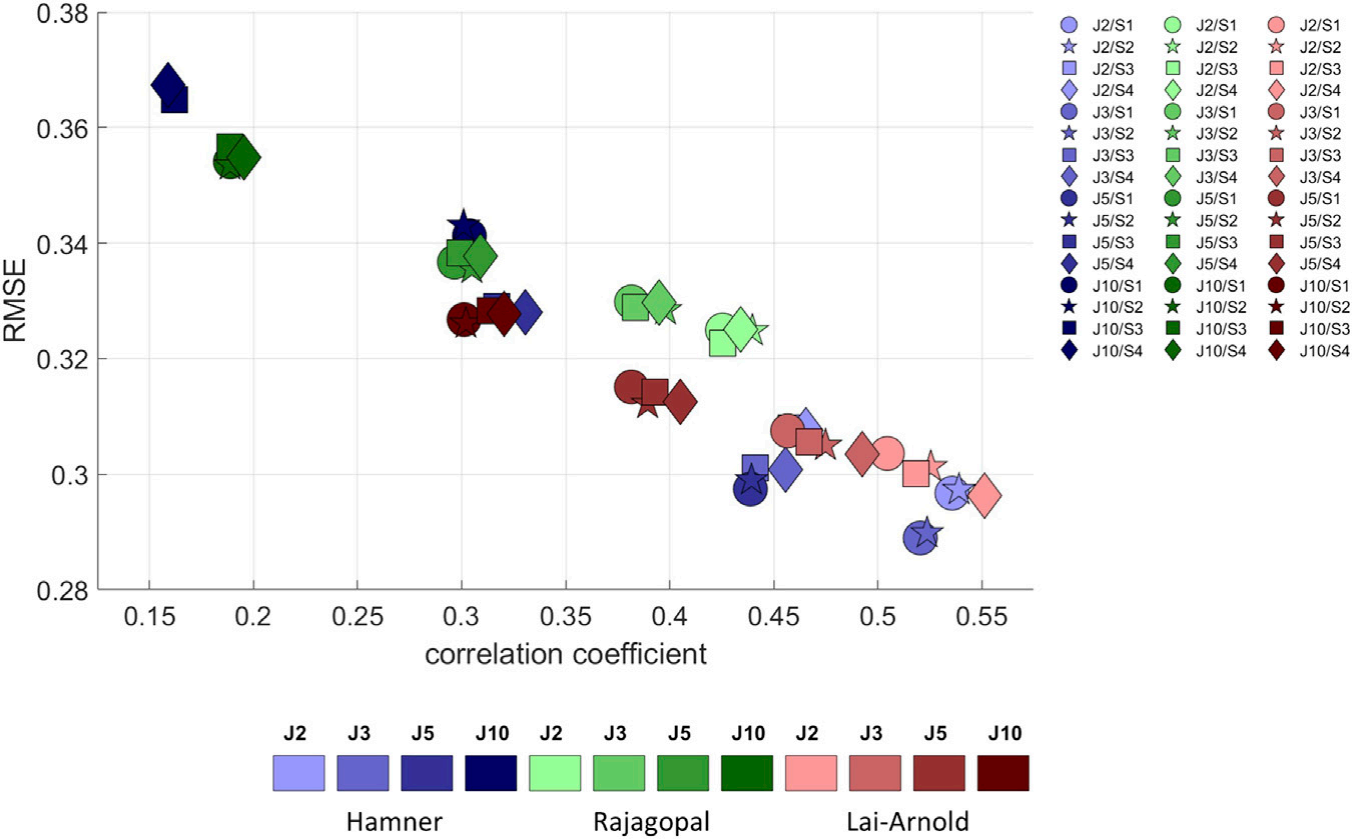
RMSE *versus* correlation coefficient (r) between normalized EMGs and estimated muscle excitations, averaged values among all subjects, speeds, and muscles, with performance criteria based on minimization of muscle effort to the power of 2 (J2), 3 (J3), 5 (J5), and 10 (J10), illustrated with darkening colors, and scaling variants of linear scaling of OFL and TSL and generic MIF (S1), non-linear scaling of OFL and TSL and generic MIF (S2), linear scaling of OFL and TSL and scaled MIF (S3), and non-linear scaling of OFL and TSL and scaled MIF (S4).

For all models and scaling variants, excitation patterns estimated with performance criterion J2 agreed better with EMG, demonstrated by lower RMSE and higher correlation coefficients, than excitation patterns estimated by with performance criteria with higher powers (Figure 3). Muscle excitations estimated with Hamner, and Lai-Arnold models agreed better with EMG than those with the Rajagopal model when combined with performance criterion J2. Among the models, performance criterion had the largest influence on estimated muscle excitations with the Hamner model. Estimated muscle activation with the Lai-Arnold model agreed better with EMG than with Rajagopal model for all performance criteria.

The scaling variant had a smaller influence on agreement between estimated muscle excitations and EMG than the model or performance criterion. With Hamner model, regardless of performance criterion, the agreement between estimated muscle excitations and EMG was better when MIF was not scaled (Supplementary Figure S1). With the Lai-Arnold model, the agreement between estimated muscle excitations and EMG was best with non-linearly scaled OFL and TSL and scaled MIF (S4). With the Rajagopal model, the scaling variant had little influence on the agreement between estimated muscle excitations and EMG.

**FIGURE 4:**
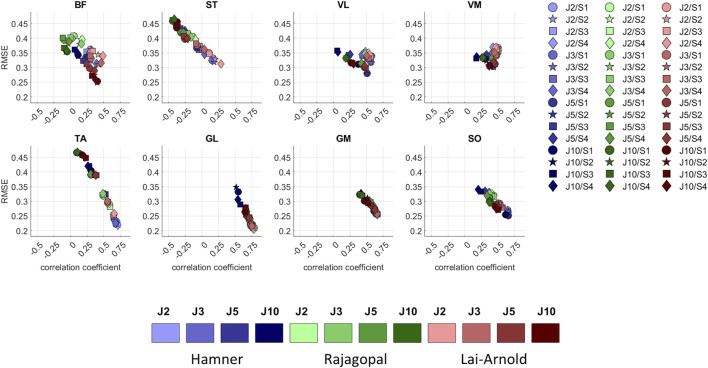
RMSE *versus* correlation coefficient (r) between normalized EMGs and estimated muscle excitations in 8 muscles, averaged values among all subjects and speeds, with performance criteria based on minimization of muscle effort to the power of 2 (J2), 3 (J3), 5 (J5), and 10 (J10), illustrated with darkening colors, and scaling variants of linear scaling of OFL and TSL and generic MIF (S1), non-linear scaling of OFL and TSL and generic MIF (S2), linear scaling of OFL and TSL and scaled MIF (S3), and non-linear scaling of OFL and TSL and scaled MIF (S4).

While muscle excitation time-series agreement with EMG was higher with Hamner and Lai-Arnold models than with the Rajagopal model, no single model estimated excitation time-series with highest agreement for all muscles (Figures 1, 4, Supplementary Tables S3, S4). The best time-series agreement of TA and SO are obtained with the Hamner model and scaling variants without scaled MIF (S1 and S2) and the Lai-Arnold model with scaling method. Time-series agreement in GM and GL were similar in all models with any scaling variant. Time-series agreement in VL and VM were similar with Rajagopal and Lai-Arnold models and any scaling variant, and with the Hamner model and scaling variants without scaled MIF (S1 and S2). Time-series agreement in ST and BF was poor in all models (Figures 1, 4).

**FIGURE 5:**
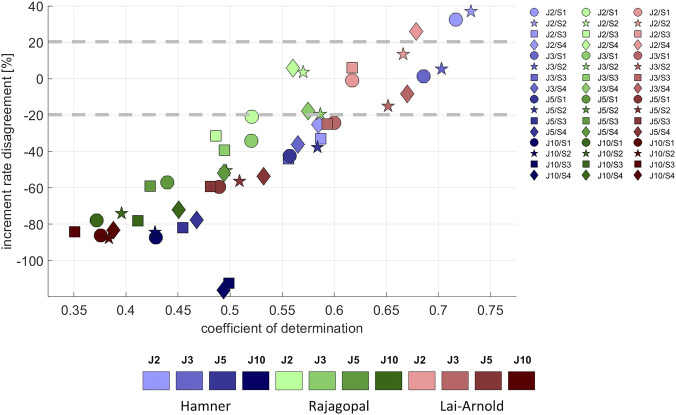
Increment rate disagreement between experimental and estimated excitations *versus* the coefficient of determination, averaged values among all subjects, speeds and muscles, with performance criteria based on minimization of muscle effort to the power of 2 (J2), 3 (J3), 5 (J5), and 10 (J10), and scaling variants with linear scaling of OFL and TSL and generic MIF (S1), non-linear scaling of OFL and TSL and generic MIF (S2), linear scaling of OFL and TSL and scaled MIF (S3), and non-linear scaling of OFL and TSL and scaled MIF (S4). The best estimates can be considered to lie approximately +/- 20%.

Average EMG increased approximately linearly with increasing walking speed (Supplementary Figure S2), as did estimated muscle excitation in all models, scaling variants and performance criteria (Supplementary Figure S3). The performance criterion had the largest influence on the agreement between the predicted and observed excitation/speed increments, with performance criteria J2 and J3 resulting in the best agreement (lowest increment rate disagreement and highest coefficient of determination with EMG increment rate) in all models (Figure 5). Increment rate agreement with the Hamner model was more influenced with MIF scaling (S3 and S4) than with OFL and TSL scaling (S1 and S2), but agreement was not notably better in any model with any specific scaling variant.

In estimated excitation/speed increments in individual muscles from all models, the lowest increment rate disagreement was observed with performance criterion J2, followed by J3. Increment rate agreement varied among model combinations and muscles; no single model combination best estimated increment rate for all muscles (Supplementary Table S5). The increment rates in SO were best estimated with the Rajagopal model, in GM and VM with the Lai-Arnold model, and in GL and TA with the Hamner model using scaling variants without scaled MIF (S1 and S2). The increment rate of VL was highly influenced by scaling variant. Increment rates in ST and BF were not estimated accurately with any model or scaling variant. The Hamner model with scaling variants without scaled MIF (S1 and S2) tended to overestimate increment rates in GM, VM, and VL and to underestimate them in SO. The Rajagopal model tended to underestimate increment rates in GM, GL, and TA and to overestimate them in VL. The Lai-Arnold model tended to underestimate increment rates in SO, GL, and TA.

**FIGURE 6:**
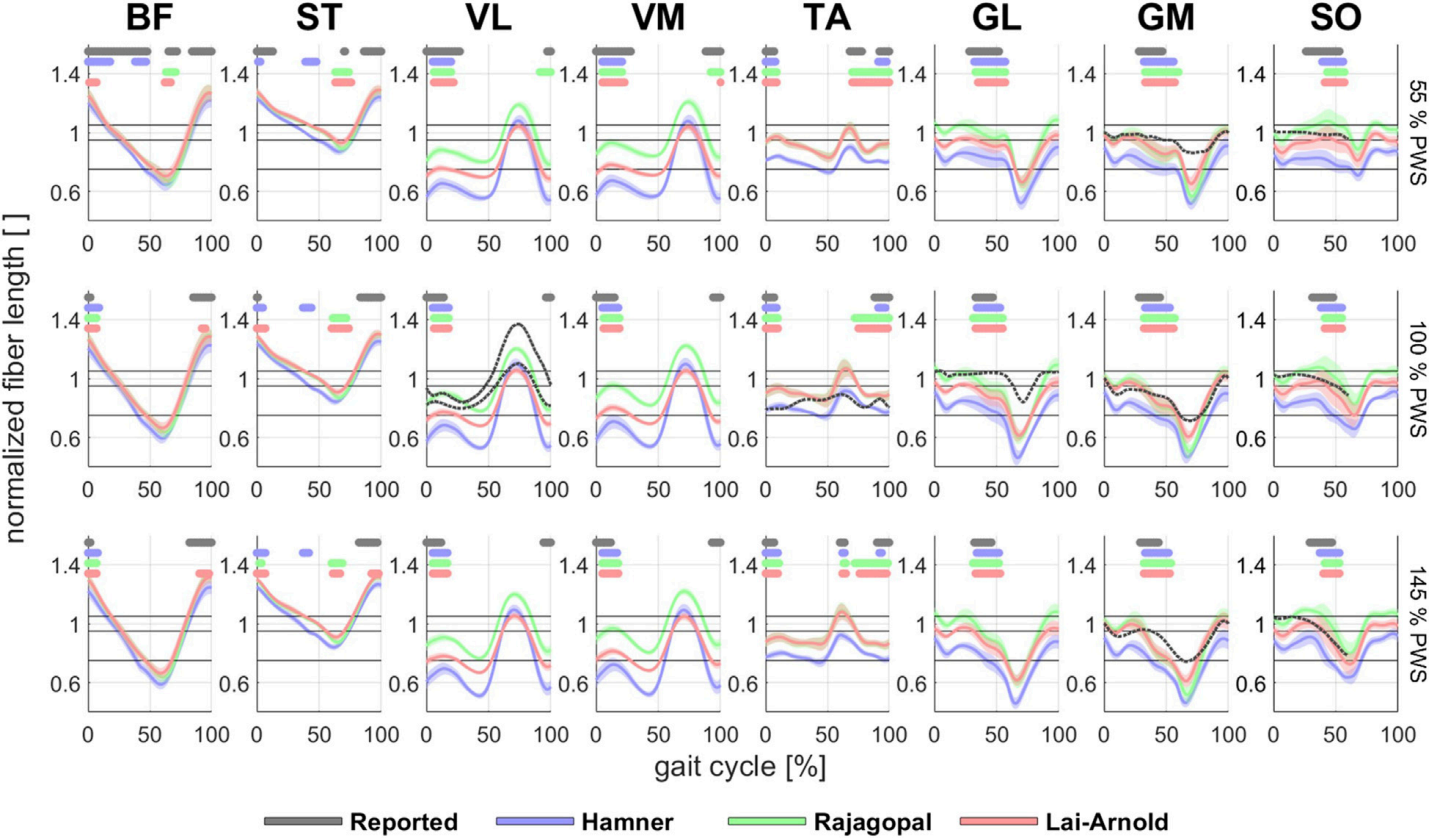
Estimated normalized fiber length (average +/- 1 SD of all subjects) in 8 muscles with performance criterion J2 and scaling variant S1 at walking speeds of 55%, 100% and 145% PWS. Reported experimental normalized fiber lengths of SO (Lai et al., 2015), GM (Farris and Sawicki, 2012), GL (Farris and Raiteri, 2017), and VL (Chleboun et al., 2007; Bohm et al., 2018) are shown. Experimental fiber lengths were normalized based on average values reported from a muscle architecture data set (Ward et al., 2009) if experimental studies did not provide normalized values. Horizontal lines above time series indicate on/off timing for EMG and each model. Dashed horizontal lines indicate operating ranges between steep and shallow ascending limb (lower), shallow ascending limb and plateau (middle), and plateau and descending limb (upper).

Computed muscle fiber lengths varied among the different models and scaling variants. All models with linearly scaled OFL and TSL (S1) estimated different fiber lengths, largely visible as length offsets but similar length changes. Fiber lengths estimated by the Rajagopal model agreed best with reported experimental findings in VL, GL, GM, and SO (Figure 6). MTU actuators of VL and GM were active during shallow ascending limb region, and SO, during shallow ascending limb and plateau regions, which coincided with reported values in the literature. Fiber lengths of BF, ST and TA estimated by Rajagopal, and Lai-Arnold models operated at similar regions. The Hamner model estimated shorter muscle fibers than the other models in all muscles. The most notable fiber length differences between models were observed in the VL, VM, and SO, where the muscles contracted at distinct operating ranges within the gait cycle. All models overestimated the excursion of the fiber lengths in GL and GM (especially the Rajagopal model), none of them estimated an isometric contraction in the VL during stance (Chleboun et al., 2007; Bohm et al., 2018), and none of them estimated the TA fiber length nor length changes well. In addition, slight differences were observed when OFL and TSL were linearly (S1) and non-linearly (S2) scaled (Supplementary Figure S4). For example, larger GL an GM fiber excursion was estimated with non-linear vs. linear OFL and TSL scaling, associated with higher MTU compliance (Table 1).

## Discussion

We have performed an extensive sensitivity analysis to investigate the influence of musculoskeletal models, scaling variants and optimization criteria for solving muscle redundancy, as well on the interactions between them, on estimation of muscle excitation as time series and over a range of walking speed, specifically how well they agree with experimentally observed EMG signals from a group of able-bodied adults. For each gait cycle, 48 different model combinations were computed and analyzed. We have illustrated which combination(s) of musculoskeletal model, MTU scaling variant, and performance criterion estimates 1) muscle excitations as time series and as excitation/speed increments that agree best with observed EMGs, and 2) muscle fiber lengths that best agree with reported experimentally-measured fiber lengths. We found that the best excitation on/off timing agreement was estimated with performance criterion J2 in all models The best time-series agreement was estimated using a performance criterion J2 with the Lai-Arnold model and any scaling variant, and with the Hamner model and scaling variant without scaled MIF (S1 and S2). The best excitation/speed increment rate agreement was estimated with performance criterion J2, followed by, J3 in all models. No single models best estimated muscle excitation time series or increment rates for all muscles; the agreement varied among model combinations. The Rajagopal model predicted fiber lengths and length change patterns that most closely resembled those reported in literature, though some discrepancies were observed in all models.

Among performance criteria, muscle excitations across walking speeds were estimated best when minimizing muscle effort to a power of 2 (J2) than to higher powers, which corroborates findings in a recent study evaluating the effect of performance criterion for walking at a self-selected speed (Zargham et al., 2019). Ackermann and van den Bogert proposed that neural commands were estimated better by minimizing muscle effort to the power of 10 (Ackermann and van den Bogert, 2010), which is practically equivalent to a min/max criterion (Rasmussen et al., 2001). The results in the present study contradict these findings to some degree; for instance, we observed that a higher exponent produced higher excitations during the swing phase, which did not correspond to observed EMGs time-series profiles (Supplementary Figure S1). This contradiction might be due in part to model sophistication; Ackermann and van den Bogert used a simplified 2D musculoskeletal model with eight muscles and estimated ground reaction force and kinematics, whereas we used 3D musculoskeletal models with a large number of muscles and prescribed inverse kinematics and inverse dynamics solutions, similar to Zargham et al. (Zargham et al., 2019). In addition, a simulation study from Arnold et al. (Arnold et al., 2013) reported good on/off timing estimations using computed muscle control (Thelen et al., 2003), with performance criterion of minimization of muscle effort to the power of 2. Our findings therefore support a recommendation to optimize for muscle redundancy base on a minimization of muscle effort to a power of 2 or perhaps 3 to estimate muscle excitations over walking speeds when kinematics and reaction forces are known.

Our findings do not clearly support any single MTUs scaling variant. It is unlikely that simple methods to scale MTUs accurately represent muscle architecture since muscle properties do not correlate well with anthropometric measurements, but scaling may improve muscle excitation estimation. Medical imaging studies using MRI have shown that muscle volume correlates reasonably well with height, and with body mass in young, healthy adults (Handsfield et al., 2014; Charles et al., 2019b). However, OFL did not correlate well with leg length (Ward et al., 2009; Charles et al., 2019b). It is hard to scale based on anthropometric measurements, and its accuracy will affect MIF estimation (Eq. 1). Specific tension at each muscle (Maganaris et al., 2001) is not well known. In this regard, we found that no single scaling method improved estimated muscle excitations, i.e., agreement with EMGs, for all muscles and all models, though some degree of personalization could result in better estimations of muscle excitation. Using non-linear scaling of OFL and TSL and scaled MIF (S4), the Lai-Arnold model had better time-series estimations (Figure 3), and the Rajagopal model had better increment rate agreement (Figure 5), than without scaling. Despite this improvement, MIF scaling was based on estimating observations in young adults (Handsfield et al., 2014). Thus, these findings should not be extrapolated to other populations, such as children or persons with neuromuscular disorders.

Estimations of muscle excitations with the Hamner model were not improved with non-linear vs. linear OFL and TSL scaling and were even worse when MIF was scaled. Good estimations of on/off timing were achieved at different speeds in most muscles, but the magnitudes (Figure 1) were considerably higher than those estimated by other models, and normalized fiber lengths were shorter than those from reported experimental observations (Figure 6). Substantially high muscle excitations can be attributable to low MIF values and shorter normalized fiber lengths, which also disagreed with their corresponding values derived from experimental observations. The Hamner model adopted MIF values from the model developed by Delp et al. (Delp et al., 1990) with some modifications. MIF was computed based on physiological cross-sectional area from both cadavers (Delp et al., 1990) as in the Delp model and also healthy individuals (Carhart, 2000), and incorporated scaling factors that varied between muscles. These tuned values were substantially lower than MIF values derived from a single dataset based on muscle volume in healthy adults (Handsfield et al., 2014), which the Rajagopal and Lai-Arnold model incorporate. The scaled MIF values in the Hamner model were slightly smaller than those in the Rajagopal and Lai-Arnold models, except in VL and VM, where incidentally the lowest time-series and excitation/speed increment agreement were found. The scaled MIF in VL from the Hamner model was even higher than those reported from maximal isometric knee extension (Bohm et al., 2018). Also, normalized fiber lengths estimated from musculoskeletal modelling are typically lower than those measured experimentally with ultrasound (Figure 6). The combination of low MIF values and short normalized fiber lengths produced a low force-generation capacity, which, for instance, resulted in an estimation that the SO reaches full excitation at high walking speeds (Figure 1).

Some disagreements between estimated muscle excitations and EMGs were common across models, scaling methods, and performance criteria, which indicates that other modeling simplifications or experimental limitations might underlie these discrepancies. Estimated plantarflexor excitation was delayed with respect to EMG, which may be attributable to inaccuracies in representing MTU parameters and activation dynamics. Delabastita et al. demonstrated better temporal agreement with observed excitations when OFL, TSL, PA, and tendon stiffness parameters were personalized using ultrasound information and recorded EMGs (Delabastita et al., 2020). Also, activation dynamics, rate encoding, and motor unit recruitment were simplified into a first-order differential model with the same activation and deactivation time constants for all muscles and all subjects, whereas these parameters have been reported as functions of muscle fiber-type composition (Winters, 1995) and age (Thelen, 2003). The electromechanical delays, i.e. the duration between muscle twitch and force production, also depends on several factors such as muscle fiber-type composition, firing rate dynamics, and viscoelastic properties of the muscle and connective tissue (De Luca, 1997), none of which were modeled in the current study.

All simulations estimated lower co-activation compared to observed EMGs, which might be related to the optimization and performance criteria. MTU controls generally minimize overall muscle effort, whereas co-activation is a likely response to increase stability in dynamics tasks (Oomen et al., 2015). A high exponent in the performance criterion increased co-activation (Supplementary Figure S1) but resulted in excitation estimates that agreed poorly for all models and scaling variants (Figures 3, 5). Modeling approaches in which MTU controls are solved by minimizing muscle effort and including feedforward and feedback control to account for sensory and motor noise (Van Wouwe et al., 2022) or by regulating mechanical impedance at the joints (Shourijeh and Fregly, 2020), better represent intrinsic motor coordination characteristics; these approaches are more likely to better estimate co-activation than minimizing muscle effort with a high exponent.

Some disagreements between estimated fiber lengths and reported experimentally-measured fiber lengths in literature are also common across models, scaling variants, and performance criteria, and are related to the models’ simplified muscle geometry. The Rajagopal model best estimated fiber lengths in VL, GL, GM, and SO, though the estimated fiber excursions were larger than those reported in the literature. A major reason for this is that modelling a muscle as unidimensional actuators does not provide a sufficient representation to capture muscle contraction throughout the volume; Aeles et al. report variations in fiber length and length changes in different fascicles of the same muscle (Aeles et al., 2022). Similarly, muscle attachments are modelled as points, whereas they in reality are surface areas. A study compared a three-dimensional fiber geometry and a lumped parameter model and showed that lumped parameter models overestimated fiber excursion (Blemker and Delp, 2006). Another factor involved in overestimating muscle excursion might be the tendon stiffness value. Modeling tendon as compliance seems a critical assumption as its influences fiber length excursion during walking (Lichtwark and Wilson, 2007; Lai et al., 2015). This study adopted the value of the Achilles tendon’s stiffness from a previous study (Delabastita et al., 2020) which allowed us to estimate similar fiber length patterns in GL, GM, and SO similar to reported with experimental observations. Nonetheless, it was assumed that triceps surae muscles shared the same normalized stiffness (equal to 15), which affected each model differently as its absolute (non-normalized) value depended upon MIF and TSL and, therefore, varied among muscles.

Also, other modeling simplifications have likely influenced fiber length estimations. For instance, patellar and quadriceps tendons were not modeled independently in any of the models, whereas they have been demonstrated to have different functions and mechanical properties (Sprague et al., 2019). In addition, measuring skeletal motion in the foot requires multi-segmental foot models, which in turn influence fiber lengths in triceps surae muscles (Zandbergen et al., 2020). By incorporating a more detailed description of patellar and quadriceps tendons, and bone foot geometry, it would be expected to capture muscle paths better and improve the representativeness of MTU mechanics in generic models.

Further limitations in studying the validity of musculoskeletal models should be noted. First, evaluation of estimated muscle excitations as time series largely consisted of comparing them to observed surface EMGs, but surface EMG signals in turn are sensitive to electrode placement, muscle fatigue, crosstalk from nearby muscles, and data post-processing methods. Furthermore, surface EMGs were measured with one bipolar sensor per muscle. It has been reported that EMG signals may vary among different regions of the same muscle (Vigotsky et al., 2018). In addition, modelled MTU time-dependent behavior will change if a different simulation approach is adopted, for instance, in dynamically consistent or in EMG-informed simulations. Thus, the results of these studies should be interpreted in the context of the modelling approach.

In terms of the generalizability of the results, the evaluation was performed using a direct collocation dynamic optimization which represents some advantages with respect to other algorithms used to solve muscle redundancies such as static optimization and computed muscle control (Thelen et al., 2003). For instance, the tendon deformation is neglected in static optimization, which might not be an appropriate assumption in large tendons such as the Achilles tendon (Lai et al., 2015) and patellar tendon (Bohm et al., 2018), and computed muscle control is sensitive to mass and inertia properties, as well as the initial time of the simulation (Wesseling et al., 2014). In contrast, the algorithm developed by DeGroote et al. incorporates tendon compliance and has a more robust formulation of the dynamic optimization than using direct shooting methods (De Groote et al., 2016). In addition, the software toolkit OpenSim Moco has been recently implemented in OpenSim, and uses direct collocation methods similar to the algorithm used in this study (Dembia et al., 2020). In this regard, our results should be compatible with the ones provided by OpenSim Moco when the inverse kinematic and inverse dynamic solutions are prescribed.

Future work to improve biofidelity of estimated muscle excitations from generic musculoskeletal models may include more detailed descriptions of muscle and bone geometry and paths and incorporate more experimental observations for their validation. We also conclude that evaluating biofidelity with on/off timing agreement and reliance on reserve actuators is insufficient to characterize MTU mechanics, as different musculoskeletal models, scaling methods, and optimization criteria estimate different fiber length patterns with good on/off timing agreement and no dependency in reserve actuators. As such, caution must be taken in interpreting estimations across different load conditions, for instance, the prediction of metabolic energetics within different walking speeds, as estimates might be misleading if MTU time-dependent behavior such as muscle excitations or fiber lengths are not adequately validated.

Finally, the Lai-Arnold model using non-linear scaling of OFL and TSL and scaled MIF with a performance criterion based on muscle effort squared better captured muscle excitations among all the variants evaluated in this study. Interestedly, the Rajagopal model, using either linear or non-linear scaling of OFL and TSL and scaled MIF with a performance criterion based on muscle effort squared, better captured fiber lengths compared to reported data in the literature. The Lai-Arnold model was derived from the Rajagopal model, and similarities were therefore expected. However, the modifications performed by Lai et al. (Lai et al., 2018) in the original formulation of the Rajagopal model improved muscle excitation estimations across walking speeds, particularly in the BF, and ST (Figures 1, 4), but yielded a higher disagreement of fiber lengths, especially in the VL. Thus, it is suggested that, among all the variants evaluated in this study, the Lai-Arnold model using non-linear scaling of OFL and TSL and scaled MIF with performance criterion based on muscle effort squared might be preferred if muscle excitation estimates are the outcomes of interest, but caution is advised in interpreting fiber length estimations. On the other hand, the Hamner model had the lowest biofidelity, which also led to the low force-generation capacity of the MTU actuators compared to other models. Therefore, the accuracy of the estimations of the muscle-tendon dynamics, particularly at high walking speeds, should be considered in the context of its application.

## Conclusion

In this study, three generic musculoskeletal models, four scaling variants, and four performance criteria based on muscle effort minimization were performed to examine how they influence estimated muscle excitations as time series, based on experimental data at different walking speeds. Interactions between them were analyzed to determine which modelling combination estimated muscle excitations and fiber lengths that best agreed with observed EMGs at different walking speeds. We found best on/off timing and excitation/speed increment rates agreement with the performance criterion of minimized muscle effort to the power of 2 in all models and scaling methods, compared to criteria with higher powers. Among models and scaling variants, we found best time-series agreements with the Hamner model without scaled MIF and with the Lai-Arnold model with non-linearly scaled OFL and TSL and scaled MIF. Overall, muscle excitations were best estimated with the Lai-Arnold model, but fiber lengths best agreed with previously reported experimentally-measured fiber lengths with the Rajagopal model. Despite moderately good estimated excitations in most muscles, the Hamner model required higher reserve actuators and estimated fiber lengths that agreed least with those reported in the literature. Common disagreements with EMG were observed in all model combinations, such as excitation delays and underestimated co-activation, which point to both model simplifications and to human motor behavior complexity.

## Data Availability

The raw data supporting the conclusion of this article will be made available by the authors, without undue reservation.
