# Supplementary material for: Evaluation of musculoskeletal models, scaling methods, and performance criteria for estimating muscle excitations and fiber lengths across walking speeds
Source: Front Bioeng Biotechnol. 2022 Oct 6;10:1002731. doi: 10.3389/fbioe.2022.1002731 (PMC9583830; doi:10.3389/fbioe.2022.1002731)
Supplement: Supplementary file 1 [file DataSheet1.pdf]

Supplementary Material

1 Supplementary Tables

Supplementary Table 1. Average and SD of OFL [cm] in 8 muscles as reported by Ward et al. (Ward et al. 2009). The table indicates which model(s) and scaling variant estimated OFL that was outside of the reported range, defined as average +/- SD.

MUSCLE	MEAN	SD	SCALED MODELS OUTSIDE THE RANGE	
			OFL and TSL linearly scaled	OFL and TSL non-linearly scaled
BF	9.76	2.62		
ST	19.30	4.12		
VL	9.94	1.76		HAMNER (underestimated)
VM	9.68	2.30		
TA	6.83	0.79	HAMNER (overestimated)	HAMNER (overestimated)
GL	5.88	0.95	LAI-ARNOLD (overestimated)	
GM	5.10	0.98	HAMNER (overestimated)	
SO	4.40	0.99		

Supplementary Table S2. Number of gait cycles (out of 21, hyphen indicates n=0) in which reserve actuators at each joint were higher than 2.5 N·m, and their peak magnitudes [N·m] in brackets.

MODEL	DOF	J2				J3				J5				J10			
		S1	S2	S3	S4	S1	S2	S3	S4	S1	S2	S3	S4	S1	S2	S3	S4
Hamner	Ankle	2 [2.8]	3 [3.1]	-	-	2 [2.8]	3 [3.2]	-	-	1 [2.7]	3 [3.3]	-	-	1 [2.6]	3 [3.4]	-	-
	Knee	1 [2.7]	-	-	-	1 [2.6]	-	-	-	-	-	-	-	1 [2.5]	-	-	-
	Hip-S	7 [18.5]	8 [19.3]	-	-	7 [18.6]	9 [19.4]	-	-	7 [18.6]	9 [19.5]	-	-	7 [18.6]	9 [19.5]	-	-
	Hip-F	-	-	-	-	-	-	-	-	-	-	-	-	-	-	-	-
	Hip-T	1 [3.9]	2 [4.1]	-	-	1 [3.8]	2 [4.1]	-	-	1 [3.8]	1 [4.1]	-	-	1 [3.9]	1 [4.2]	-	-
Rajagopal	Ankle	-	-	-	-	-	-	-	-	-	-	-	-	-	-	-	-
	Knee	-	-	-	-	-	-	-	-	-	-	-	-	-	-	-	-
	Hip-S	1 [18.0]	1 [2.6]	2 [21.8]	-	2 [18.0]	1 [3.1]	2 [21.8]	1 [2.6]	2 [18.0]	1 [3.2]	2 [21.8]	1 [2.7]	2 [18.0]	1 [3.2]	2 [21.8]	-
	Hip-F	-	-	-	-	-	-	-	-	-	-	-	-	-	-	-	-
	Hip-T	-	-	-	-	-	-	-	-	-	-	-	-	-	-	-	-
Lai-Arnold	Ankle	-	-	-	-	-	-	-	-	-	-	-	-	-	-	-	-
	Knee	-	-	-	-	-	-	-	-	-	-	-	-	-	-	-	-
	Hip-S	-	-	-	1 [2.6]	-	-	-	1 [3.0]	-	-	-	1 [3.0]	-	-	-	1 [3.1]
	Hip-F	-	-	-	-	-	-	-	-	-	-	-	-	-	-	-	-
	Hip-T	-	-	-	-	-	-	-	-	-	-	-	-	-	-	-	-

Supplementary Table S3. Average and SD of correlation coefficient between normalized EMGs and estimated muscle excitations among all subjects and speeds in Hamner (H), Rajagopal (R), and Lai-Arnold (L) model, for performance criteria J2, J3, J5, and J10 and scaling methods S1, S2, S3, and S4.

MUSCLE		J2				J3				J5				J10			
		S1	S2	S3	S4	S1	S2	S3	S4	S1	S2	S3	S4	S1	S2	S3	S4
BF	H	0.30 [0.20]	0.32 [0.19]	0.24 [0.22]	0.30 [0.22]	0.29 [0.20]	0.31 [0.19]	0.29 [0.22]	0.39 [0.22]	0.23 [0.18]	0.25 [0.18]	0.19 [0.21]	0.30 [0.21]	0.11 [0.19]	0.11 [0.19]	0.06 [0.20]	0.10 [0.20]
	R	0.06 [0.24]	0.16 [0.26]	0.04 [0.24]	0.17 [0.26]	0.02 [0.23]	0.14 [0.27]	0.00 [0.22]	0.17 [0.27]	-0.10 [0.18]	-0.03 [0.20]	-0.11 [0.17]	-0.02 [0.20]	-0.05 [0.22]	-0.07 [0.19]	-0.10 [0.18]	-0.06 [0.16]
	L	0.31 [0.29]	0.42 [0.26]	0.34 [0.28]	0.49 [0.26]	0.31 [0.29]	0.42 [0.28]	0.30 [0.28]	0.46 [0.28]	0.29 [0.30]	0.36 [0.29]	0.24 [0.27]	0.36 [0.27]	0.41 [0.28]	0.40 [0.27]	0.33 [0.27]	0.38 [0.26]
ST	H	0.17 [0.26]	0.20 [0.24]	-0.03 [0.21]	0.02 [0.21]	0.15 [0.24]	0.18 [0.23]	-0.05 [0.21]	0.04 [0.21]	-0.08 [0.21]	-0.07 [0.20]	-0.32 [0.14]	-0.26 [0.14]	-0.37 [0.17]	-0.36 [0.16]	-0.53 [0.13]	-0.50 [0.13]
	R	-0.13 [0.15]	-0.09 [0.17]	-0.15 [0.15]	-0.10 [0.15]	-0.21 [0.13]	-0.16 [0.15]	-0.21 [0.13]	-0.16 [0.13]	-0.32 [0.14]	-0.30 [0.15]	-0.32 [0.12]	-0.27 [0.13]	-0.44 [0.14]	-0.43 [0.15]	-0.44 [0.12]	-0.41 [0.13]
	L	0.11 [0.26]	0.20 [0.27]	0.11 [0.27]	0.28 [0.25]	-0.07 [0.19]	-0.01 [0.21]	-0.09 [0.17]	0.03 [0.17]	-0.26 [0.15]	-0.24 [0.16]	-0.27 [0.12]	-0.21 [0.13]	-0.38 [0.14]	-0.38 [0.14]	-0.39 [0.11]	-0.37 [0.13]
VL	H	0.58 [0.16]	0.58 [0.16]	0.49 [0.18]	0.48 [0.18]	0.56 [0.17]	0.56 [0.17]	0.44 [0.18]	0.43 [0.19]	0.51 [0.18]	0.50 [0.18]	0.23 [0.15]	0.25 [0.16]	0.38 [0.19]	0.38 [0.19]	0.06 [0.17]	0.07 [0.17]
	R	0.55 [0.17]	0.54 [0.17]	0.56 [0.17]	0.53 [0.17]	0.51 [0.18]	0.50 [0.17]	0.52 [0.17]	0.48 [0.18]	0.43 [0.18]	0.42 [0.18]	0.43 [0.17]	0.42 [0.16]	0.20 [0.16]	0.19 [0.17]	0.19 [0.15]	0.19 [0.15]
	L	0.56 [0.17]	0.55 [0.17]	0.56 [0.17]	0.54 [0.17]	0.52 [0.17]	0.51 [0.17]	0.53 [0.17]	0.50 [0.18]	0.47 [0.17]	0.46 [0.18]	0.49 [0.16]	0.47 [0.17]	0.27 [0.18]	0.27 [0.19]	0.29 [0.16]	0.31 [0.16]
VM	H	0.50 [0.21]	0.50 [0.21]	0.43 [0.21]	0.42 [0.21]	0.48 [0.21]	0.48 [0.21]	0.42 [0.20]	0.41 [0.20]	0.42 [0.21]	0.43 [0.21]	0.33 [0.18]	0.33 [0.19]	0.31 [0.21]	0.33 [0.21]	0.17 [0.18]	0.18 [0.19]
	R	0.50 [0.21]	0.49 [0.22]	0.51 [0.21]	0.47 [0.22]	0.48 [0.22]	0.47 [0.22]	0.49 [0.21]	0.45 [0.22]	0.42 [0.22]	0.41 [0.22]	0.42 [0.21]	0.41 [0.22]	0.27 [0.22]	0.26 [0.22]	0.27 [0.21]	0.27 [0.21]
	L	0.49 [0.21]	0.48 [0.22]	0.48 [0.21]	0.46 [0.22]	0.48 [0.21]	0.46 [0.22]	0.47 [0.21]	0.44 [0.22]	0.45 [0.21]	0.44 [0.22]	0.45 [0.21]	0.43 [0.21]	0.37 [0.21]	0.36 [0.22]	0.38 [0.20]	0.37 [0.21]
TA	H	0.69 [0.11]	0.68 [0.11]	0.71 [0.10]	0.71 [0.10]	0.65 [0.10]	0.64 [0.10]	0.68 [0.09]	0.68 [0.09]	0.49 [0.15]	0.48 [0.15]	0.53 [0.13]	0.52 [0.13]	0.31 [0.19]	0.30 [0.19]	0.28 [0.20]	0.26 [0.20]
	R	0.59 [0.15]	0.59 [0.15]	0.59 [0.15]	0.59 [0.16]	0.49 [0.18]	0.49 [0.18]	0.50 [0.18]	0.49 [0.19]	0.31 [0.23]	0.31 [0.22]	0.33 [0.22]	0.30 [0.22]	0.09 [0.28]	0.09 [0.27]	0.12 [0.26]	0.10 [0.24]

	L	0.65 [0.13]	0.65 [0.12]	0.65 [0.13]	0.66 [0.13]	0.56 [0.15]	0.55 [0.14]	0.57 [0.15]	0.56 [0.15]	0.37 [0.19]	0.37 [0.18]	0.39 [0.19]	0.38 [0.19]	0.18 [0.25]	0.17 [0.24]	0.23 [0.22]	0.22 [0.21]
GL	H	0.75 [0.09]	0.75 [0.09]	0.75 [0.09]	0.75 [0.09]	0.73 [0.09]	0.72 [0.09]	0.72 [0.10]	0.71 [0.09]	0.67 [0.09]	0.66 [0.09]	0.66 [0.11]	0.65 [0.11]	0.55 [0.11]	0.52 [0.10]	0.58 [0.12]	0.54 [0.13]
	R	0.78 [0.08]	0.78 [0.08]	0.78 [0.08]	0.78 [0.09]	0.76 [0.09]	0.75 [0.09]	0.76 [0.09]	0.76 [0.10]	0.71 [0.11]	0.71 [0.10]	0.71 [0.11]	0.72 [0.10]	0.66 [0.11]	0.66 [0.11]	0.66 [0.11]	0.67 [0.11]
	L	0.76 [0.09]	0.76 [0.09]	0.77 [0.09]	0.77 [0.10]	0.74 [0.09]	0.73 [0.09]	0.75 [0.09]	0.75 [0.10]	0.69 [0.10]	0.69 [0.10]	0.70 [0.11]	0.71 [0.10]	0.65 [0.11]	0.65 [0.11]	0.65 [0.11]	0.66 [0.11]
GM	H	0.66 [0.12]	0.65 [0.12]	0.66 [0.10]	0.66 [0.10]	0.64 [0.11]	0.63 [0.12]	0.62 [0.11]	0.62 [0.11]	0.61 [0.11]	0.60 [0.12]	0.56 [0.13]	0.55 [0.12]	0.54 [0.12]	0.51 [0.12]	0.44 [0.13]	0.42 [0.13]
	R	0.62 [0.12]	0.61 [0.12]	0.63 [0.12]	0.62 [0.12]	0.59 [0.13]	0.59 [0.13]	0.61 [0.12]	0.60 [0.13]	0.54 [0.14]	0.54 [0.14]	0.56 [0.14]	0.57 [0.14]	0.39 [0.17]	0.40 [0.16]	0.40 [0.17]	0.43 [0.17]
	L	0.63 [0.11]	0.63 [0.11]	0.67 [0.11]	0.66 [0.11]	0.62 [0.11]	0.62 [0.11]	0.66 [0.11]	0.66 [0.11]	0.57 [0.12]	0.57 [0.13]	0.62 [0.12]	0.63 [0.12]	0.47 [0.14]	0.47 [0.14]	0.52 [0.14]	0.52 [0.14]
SO	H	0.64 [0.17]	0.64 [0.17]	0.41 [0.25]	0.37 [0.25]	0.65 [0.16]	0.65 [0.16]	0.41 [0.24]	0.36 [0.24]	0.65 [0.15]	0.66 [0.15]	0.35 [0.24]	0.31 [0.23]	0.61 [0.16]	0.62 [0.16]	0.24 [0.23]	0.21 [0.21]
	R	0.43 [0.24]	0.43 [0.24]	0.44 [0.26]	0.41 [0.26]	0.40 [0.24]	0.41 [0.25]	0.41 [0.26]	0.37 [0.26]	0.37 [0.24]	0.38 [0.25]	0.37 [0.26]	0.34 [0.25]	0.37 [0.21]	0.40 [0.21]	0.40 [0.22]	0.37 [0.23]
	L	0.52 [0.22]	0.52 [0.22]	0.57 [0.22]	0.55 [0.22]	0.50 [0.22]	0.50 [0.23]	0.55 [0.22]	0.53 [0.22]	0.47 [0.21]	0.47 [0.22]	0.51 [0.22]	0.48 [0.22]	0.46 [0.18]	0.48 [0.19]	0.50 [0.20]	0.48 [0.20]

Supplementary Table S4. Average and SD of RMSE between normalized EMGs and estimated muscle excitations among all subjects and speeds in Hamner (H), Rajagopal (R), and Lai-Arnold (L) model, for performance criteria J2, J3, J5, and J10 and scaling methods S1, S2, S3, and S4.

MUSCLE		J2				J3				J5				J10			
		**S1**	**S2**	**S3**	**S4**	**S1**	**S2**	**S3**	**S4**	**S1**	**S2**	**S3**	**S4**	**S1**	**S2**	**S3**	**S4**
BF	H	0.36 [0.1]	0.36 [0.1]	0.35 [0.1]	0.35 [0.1]	0.35 [0.1]	0.35 [0.1]	0.32 [0.1]	0.31 [0.1]	0.33 [0.0]	0.34 [0.0]	0.32 [0.0]	0.30 [0.0]	0.34 [0.0]	0.34 [0.0]	0.36 [0.0]	0.35 [0.0]
	R	0.41 [0.1]	0.40 [0.1]	0.41 [0.1]	0.40 [0.1]	0.40 [0.1]	0.38 [0.1]	0.40 [0.1]	0.38 [0.1]	0.39 [0.1]	0.38 [0.1]	0.40 [0.1]	0.39 [0.1]	0.35 [0.1]	0.35 [0.1]	0.37 [0.1]	0.36 [0.1]
	L	0.36 [0.1]	0.34 [0.1]	0.36 [0.1]	0.34 [0.1]	0.33 [0.1]	0.31 [0.1]	0.34 [0.1]	0.32 [0.1]	0.30 [0.1]	0.27 [0.1]	0.31 [0.1]	0.29 [0.1]	0.25 [0.1]	0.25 [0.0]	0.27 [0.1]	0.26 [0.0]
	H	0.33 [0.1]	0.33 [0.1]	0.37 [0.1]	0.36 [0.1]	0.32 [0.0]	0.32 [0.0]	0.36 [0.0]	0.34 [0.0]	0.37 [0.0]	0.37 [0.0]	0.43 [0.0]	0.43 [0.0]	0.46 [0.0]	0.46 [0.0]	0.51 [0.1]	0.51 [0.1]

ST	R	0.40 [0.0]	0.39 [0.0]	0.41 [0.0]	0.40 [0.0]	0.41 [0.0]	0.40 [0.0]	0.42 [0.0]	0.40 [0.0]	0.42 [0.0]	0.42 [0.0]	0.42 [0.0]	0.42 [0.0]	0.46 [0.0]	0.46 [0.0]	0.46 [0.0]	0.46 [0.0]
	L	0.35 [0.1]	0.33 [0.1]	0.35 [0.1]	0.31 [0.1]	0.38 [0.0]	0.36 [0.0]	0.38 [0.0]	0.36 [0.0]	0.40 [0.0]	0.39 [0.0]	0.41 [0.0]	0.40 [0.0]	0.43 [0.0]	0.44 [0.0]	0.44 [0.0]	0.45 [0.0]
VL	H	0.34 [0.1]	0.34 [0.1]	0.35 [0.1]	0.35 [0.1]	0.32 [0.1]	0.32 [0.1]	0.34 [0.1]	0.34 [0.1]	0.28 [0.0]	0.28 [0.0]	0.34 [0.0]	0.35 [0.0]	0.31 [0.0]	0.31 [0.0]	0.36 [0.0]	0.35 [0.0]
	R	0.34 [0.1]	0.35 [0.1]	0.33 [0.1]	0.35 [0.1]	0.33 [0.1]	0.34 [0.1]	0.33 [0.1]	0.35 [0.1]	0.31 [0.1]	0.31 [0.0]	0.31 [0.1]	0.32 [0.0]	0.33 [0.0]	0.33 [0.0]	0.33 [0.0]	0.33 [0.0]
	L	0.34 [0.1]	0.34 [0.1]	0.34 [0.1]	0.34 [0.1]	0.33 [0.1]	0.33 [0.1]	0.33 [0.1]	0.34 [0.1]	0.30 [0.0]	0.30 [0.0]	0.30 [0.0]	0.30 [0.0]	0.32 [0.0]	0.31 [0.0]	0.31 [0.0]	0.31 [0.0]
VM	H	0.37 [0.1]	0.37 [0.1]	0.37 [0.1]	0.37 [0.1]	0.34 [0.1]	0.34 [0.1]	0.35 [0.1]	0.35 [0.1]	0.30 [0.1]	0.30 [0.1]	0.34 [0.0]	0.34 [0.0]	0.33 [0.1]	0.32 [0.1]	0.33 [0.0]	0.34 [0.0]
	R	0.36 [0.1]	0.37 [0.1]	0.36 [0.1]	0.37 [0.1]	0.35 [0.1]	0.36 [0.1]	0.34 [0.1]	0.36 [0.1]	0.33 [0.1]	0.33 [0.1]	0.33 [0.1]	0.33 [0.1]	0.33 [0.1]	0.34 [0.1]	0.33 [0.1]	0.33 [0.1]
	L	0.36 [0.1]	0.36 [0.1]	0.36 [0.1]	0.37 [0.1]	0.34 [0.1]	0.34 [0.1]	0.34 [0.1]	0.35 [0.1]	0.31 [0.1]	0.31 [0.1]	0.31 [0.1]	0.32 [0.1]	0.31 [0.1]	0.31 [0.1]	0.31 [0.1]	0.31 [0.1]
TA	H	0.23 [0.0]	0.23 [0.0]	0.22 [0.0]	0.22 [0.0]	0.24 [0.0]	0.24 [0.0]	0.22 [0.0]	0.23 [0.0]	0.32 [0.1]	0.33 [0.1]	0.32 [0.1]	0.32 [0.1]	0.40 [0.1]	0.40 [0.1]	0.42 [0.1]	0.42 [0.1]
	R	0.29 [0.1]	0.29 [0.1]	0.28 [0.1]	0.29 [0.1]	0.32 [0.1]	0.32 [0.1]	0.32 [0.1]	0.33 [0.1]	0.39 [0.1]	0.39 [0.1]	0.39 [0.1]	0.39 [0.1]	0.47 [0.1]	0.47 [0.1]	0.46 [0.1]	0.47 [0.1]
	L	0.25 [0.0]	0.26 [0.0]	0.25 [0.0]	0.26 [0.0]	0.29 [0.1]	0.30 [0.1]	0.30 [0.1]	0.30 [0.1]	0.39 [0.1]	0.39 [0.1]	0.39 [0.1]	0.39 [0.1]	0.46 [0.1]	0.46 [0.1]	0.45 [0.1]	0.45 [0.1]
GL	H	0.21 [0.0]	0.22 [0.0]	0.22 [0.0]	0.22 [0.0]	0.22 [0.0]	0.22 [0.0]	0.23 [0.0]	0.23 [0.0]	0.25 [0.0]	0.26 [0.0]	0.25 [0.0]	0.26 [0.0]	0.33 [0.0]	0.35 [0.0]	0.29 [0.0]	0.31 [0.0]
	R	0.21 [0.0]	0.21 [0.0]	0.21 [0.0]	0.20 [0.0]	0.22 [0.0]	0.22 [0.0]	0.22 [0.0]	0.22 [0.0]	0.24 [0.0]	0.24 [0.0]	0.25 [0.0]	0.24 [0.0]	0.27 [0.0]	0.26 [0.0]	0.28 [0.0]	0.26 [0.0]
	L	0.22 [0.0]	0.22 [0.0]	0.21 [0.0]	0.21 [0.0]	0.22 [0.0]	0.22 [0.0]	0.22 [0.0]	0.22 [0.0]	0.25 [0.0]	0.25 [0.0]	0.24 [0.0]	0.24 [0.0]	0.26 [0.0]	0.26 [0.0]	0.28 [0.0]	0.28 [0.0]
GM	H	0.26 [0.0]	0.26 [0.0]	0.26 [0.0]	0.26 [0.0]	0.26 [0.0]	0.26 [0.0]	0.27 [0.0]	0.27 [0.0]	0.27 [0.0]	0.27 [0.0]	0.29 [0.0]	0.29 [0.0]	0.30 [0.0]	0.31 [0.0]	0.32 [0.0]	0.33 [0.0]
	R	0.28 [0.0]	0.28 [0.0]	0.27 [0.0]	0.27 [0.0]	0.28 [0.0]	0.28 [0.0]	0.28 [0.0]	0.28 [0.0]	0.30 [0.0]	0.30 [0.0]	0.30 [0.0]	0.29 [0.0]	0.32 [0.0]	0.32 [0.0]	0.32 [0.0]	0.32 [0.0]
	L	0.27 [0.0]	0.27 [0.0]	0.26 [0.0]	0.26 [0.0]	0.27 [0.0]	0.27 [0.0]	0.26 [0.0]	0.26 [0.0]	0.29 [0.0]	0.29 [0.0]	0.27 [0.0]	0.27 [0.0]	0.30 [0.0]	0.30 [0.0]	0.29 [0.0]	0.30 [0.0]

SO	H	0.27 [0.1]	0.27 [0.1]	0.32 [0.1]	0.33 [0.1]	0.26 [0.1]	0.26 [0.1]	0.32 [0.1]	0.33 [0.1]	0.25 [0.1]	0.25 [0.1]	0.32 [0.1]	0.33 [0.1]	0.25 [0.0]	0.25 [0.0]	0.33 [0.1]	0.34 [0.1]
	R	0.32 [0.1]	0.32 [0.1]	0.32 [0.1]	0.33 [0.1]	0.32 [0.1]	0.32 [0.1]	0.32 [0.1]	0.33 [0.1]	0.31 [0.1]	0.31 [0.1]	0.31 [0.1]	0.32 [0.1]	0.30 [0.0]	0.29 [0.0]	0.29 [0.1]	0.30 [0.1]
	L	0.29 [0.1]	0.29 [0.1]	0.28 [0.1]	0.28 [0.1]	0.29 [0.1]	0.29 [0.1]	0.28 [0.1]	0.29 [0.1]	0.29 [0.1]	0.29 [0.1]	0.28 [0.1]	0.29 [0.1]	0.28 [0.0]	0.28 [0.0]	0.27 [0.0]	0.27 [0.0]

Supplementary Table S5. Average and SD of increment rate disagreement between experimental and estimated excitation trends among all subjects in Hamner (H), Rajagopal (R), and Lai-Arnold (L) model for performance criteria J2, J3, J5, and J10 and scaling variants S1, S2, S3, and S4.

MUSCLE		J2				J3				J5				J10			
		S1	S2	S3	S4	S1	S2	S3	S4	S1	S2	S3	S4	S1	S2	S3	S4
BF	H	117.9 [173.2]	130.9 [175.5]	17.7 [125.6]	40.7 [109.4]	77.1 [160.5]	89.3 [161.4]	-3.2 [96.7]	23.5 [86.5]	32.7 [151.0]	47.0 [153.9]	-73.9 [42.1]	-60.9 [35.3]	-50.3 [107.4]	-41.2 [111.5]	-108.2 [32.6]	-112.9 [27.2]
	R	7.4 [322.3]	107.4 [177.2]	-17.1 [169.1]	143.2 [116.5]	-62.2 [99.3]	-17.3 [87.0]	-85.2 [82.4]	-0.7 [34.9]	-91.6 [19.7]	-72.0 [9.2]	-101.4 [27.5]	-71.0 [9.3]	-114.9 [31.1]	-91.8 [19.7]	-117.8 [43.8]	-81.2 [26.5]
	L	132.2 [173.0]	189.7 [154.7]	188.9 [161.0]	214.8 [178.8]	49.4 [83.9]	74.3 [76.3]	54.5 [69.0]	100.5 [106.3]	-26.9 [35.1]	-26.7 [33.8]	-35.7 [52.4]	-24.1 [46.1]	-31.3 [55.7]	-56.7 [28.8]	-39.5 [77.2]	-50.9 [44.5]
ST	H	83.9 [182.3]	97.9 [168.6]	-160.1 [48.4]	-150.4 [33.7]	-11.8 [117.3]	-0.7 [115.7]	-127.8 [48.6]	-114.3 [38.1]	-117.5 [64.2]	-99.8 [59.6]	-145.7 [46.9]	-137.3 [45.8]	-153.6 [41.8]	-137.9 [34.2]	-139.8 [26.4]	-133.2 [23.5]
	R	-91.2 [81.2]	-73.9 [85.6]	-123.9 [87.9]	-84.8 [60.3]	-42.4 [44.5]	-33.2 [48.3]	-47.3 [45.2]	-30.6 [52.6]	-71.1 [44.1]	-72.6 [27.5]	-70.9 [40.5]	-62.1 [30.7]	-92.7 [27.9]	-88.3 [16.2]	-74.4 [50.3]	-66.6 [43.1]
	L	-31.6 [152.3]	-23.5 [110.4]	-45.5 [93.9]	31.9 [90.3]	-29.5 [36.4]	-29.2 [37.3]	-41.2 [28.5]	-10.8 [57.5]	-73.5 [23.8]	-78.7 [16.3]	-69.3 [40.6]	-64.9 [30.0]	-106.0 [24.8]	-106.5 [21.7]	-85.8 [47.2]	-78.9 [39.1]
VL	H	48.0 [83.3]	49.5 [91.4]	-0.5 [97.0]	12.2 [83.9]	16.9 [69.7]	21.7 [76.4]	-24.3 [105.3]	-10.9 [86.0]	-39.2 [60.2]	-31.3 [59.1]	-105.4 [110.2]	-89.1 [101.7]	-101.2 [49.3]	-95.7 [45.6]	-172.3 [52.3]	-188.7 [61.0]
	R	29.0 [123.0]	69.8 [143.0]	21.4 [120.1]	66.0 [134.5]	17.5 [121.7]	52.0 [137.8]	14.8 [116.8]	52.1 [130.3]	-14.7 [94.0]	0.3 [101.0]	-13.5 [82.8]	-4.8 [90.5]	-48.0 [50.0]	-43.5 [50.3]	-48.5 [37.6]	-44.4 [39.9]
	L	11.2 [97.4]	37.3 [110.5]	9.8 [86.7]	36.8 [98.3]	-6.6 [90.7]	15.1 [101.7]	-13.5 [80.7]	13.0 [88.7]	-40.0 [72.8]	-24.7 [79.2]	-42.2 [61.3]	-24.4 [65.9]	-86.1 [42.4]	-82.8 [46.1]	-77.4 [35.9]	-72.1 [39.9]
VM	H	43.2 [90.3]	44.1 [95.3]	-18.7 [101.8]	-13.1 [89.9]	11.8 [80.8]	11.0 [84.1]	-49.2 [89.4]	-47.4 [80.4]	-45.2 [75.2]	-47.3 [72.1]	-99.1 [70.9]	-105.0 [58.1]	-107.3 [53.4]	-111.2 [51.4]	-149.8 [59.6]	-163.9 [46.6]
	R	4.8 [115.3]	38.2 [118.1]	-3.3 [109.6]	31.7 [111.9]	-27.2 [95.7]	0.1 [95.3]	-32.8 [87.0]	-4.8 [88.6]	-46.8 [59.6]	-29.8 [61.4]	-49.1 [51.4]	-30.8 [58.3]	-64.7 [35.6]	-57.1 [39.3]	-67.1 [30.1]	-57.7 [37.8]
	L	-13.6 [99.6]	7.1 [99.5]	-9.6 [95.3]	11.9 [94.6]	-49.0 [82.6]	-33.7 [80.7]	-44.6 [76.7]	-29.2 [74.8]	-88.5 [54.7]	-81.7 [52.5]	-83.5 [47.8]	-78.4 [48.3]	-111.5 [26.7]	-109.2 [30.0]	-114.3 [23.8]	-113.0 [29.1]
	H	4.0 [53.9]	4.9 [52.9]	-28.4 [22.9]	-28.1 [24.0]	-12.8 [46.9]	-11.4 [44.1]	-51.3 [17.5]	-50.1 [17.9]	-47.1 [39.0]	-45.7 [37.7]	-88.1 [11.1]	-87.9 [11.4]	-82.8 [28.9]	-80.9 [29.0]	-122.4 [13.9]	-122.5 [14.2]

TA	R	-41.3 [23.2]	-39.0 [22.4]	-46.9 [16.9]	-38.6 [15.7]	-61.3 [21.7]	-59.3 [22.7]	-63.3 [15.4]	-59.7 [14.3]	-91.1 [17.3]	-91.3 [17.7]	-92.4 [12.9]	-93.3 [12.3]	-115.1 [16.4]	-117.1 [17.4]	-117.4 [15.2]	-118.6 [16.0]
	L	-32.4 [30.9]	-33.3 [27.3]	-27.0 [21.3]	-29.6 [23.5]	-56.2 [24.1]	-54.6 [24.5]	-55.6 [16.0]	-53.9 [16.8]	-91.2 [16.6]	-90.0 [18.0]	-88.5 [10.9]	-88.9 [9.6]	-120.8 [15.8]	-119.8 [15.9]	-120.0 [16.6]	-120.1 [15.1]
GL	H	-7.5 [24.7]	-7.7 [21.7]	-45.9 [23.2]	-45.7 [20.1]	-32.2 [20.7]	-33.0 [18.4]	-54.0 [19.2]	-55.3 [14.8]	-53.7 [20.9]	-55.7 [18.0]	-70.5 [13.4]	-71.8 [11.2]	-72.7 [21.3]	-77.5 [17.1]	-93.4 [15.5]	-94.4 [16.6]
	R	-41.1 [21.4]	-41.0 [19.5]	-44.0 [20.3]	-34.1 [20.2]	-50.7 [17.2]	-50.4 [14.2]	-52.0 [15.6]	-47.3 [13.5]	-60.8 [17.8]	-60.8 [16.6]	-61.1 [17.6]	-57.4 [15.0]	-72.6 [22.1]	-78.9 [13.5]	-74.3 [27.4]	-76.1 [13.4]
	L	-28.7 [32.0]	-28.2 [27.4]	-25.8 [32.8]	-20.2 [27.8]	-45.8 [23.8]	-44.8 [22.3]	-46.3 [22.8]	-40.0 [22.6]	-69.6 [16.9]	-67.4 [16.9]	-69.1 [13.5]	-64.1 [15.5]	-89.4 [11.6]	-87.9 [10.6]	-90.0 [9.9]	-86.7 [10.0]
GM	H	28.0 [53.0]	31.2 [53.6]	-12.7 [44.0]	-11.2 [39.6]	15.3 [50.8]	16.9 [50.5]	-18.1 [35.8]	-19.5 [29.2]	-10.2 [46.1]	-12.8 [45.2]	-38.8 [27.6]	-40.4 [22.4]	-50.9 [43.6]	-57.0 [41.9]	-80.8 [28.2]	-80.3 [28.0]
	R	-36.9 [44.8]	-38.0 [42.3]	-36.9 [46.5]	-26.6 [44.7]	-33.7 [34.5]	-36.0 [34.5]	-32.8 [37.4]	-28.1 [38.5]	-21.3 [35.6]	-22.3 [32.5]	-22.7 [36.4]	-29.1 [27.5]	-32.1 [32.8]	-29.8 [37.8]	-29.0 [45.0]	-21.3 [38.0]
	L	4.1 [57.9]	4.7 [51.8]	8.0 [61.4]	17.2 [51.5]	-0.0 [52.1]	5.0 [50.4]	2.2 [54.7]	12.6 [50.4]	-16.1 [40.7]	-13.8 [42.0]	-16.8 [38.4]	-11.3 [38.2]	-46.0 [35.0]	-39.6 [41.0]	-49.6 [34.3]	-40.3 [36.5]
SO	H	-57.0 [50.8]	-55.6 [50.5]	-15.1 [54.8]	-6.3 [53.1]	-53.3 [43.9]	-51.5 [43.9]	-24.1 [50.1]	-14.5 [48.6]	-60.0 [33.1]	-57.0 [32.6]	-35.0 [47.5]	-28.7 [45.7]	-79.1 [24.5]	-74.5 [23.2]	-33.3 [46.1]	-33.9 [41.8]
	R	2.0 [82.8]	4.9 [73.2]	-0.7 [75.8]	-11.3 [76.5]	-14.0 [71.0]	-11.8 [63.2]	-15.9 [65.8]	-23.8 [66.2]	-59.0 [49.3]	-56.5 [46.5]	-61.7 [46.3]	-66.2 [52.0]	-82.7 [39.3]	-86.2 [38.0]	-97.4 [33.0]	-111.6 [36.1]
	L	-49.1 [58.1]	-46.5 [51.9]	-49.8 [45.9]	-55.0 [48.2]	-55.8 [52.0]	-53.4 [46.1]	-55.9 [42.3]	-59.9 [43.6]	-71.0 [47.7]	-68.3 [43.7]	-68.9 [38.7]	-73.1 [39.6]	-98.4 [41.2]	-100.0 [41.8]	-97.7 [40.7]	-104.8 [40.8]

2 Supplementary Figures

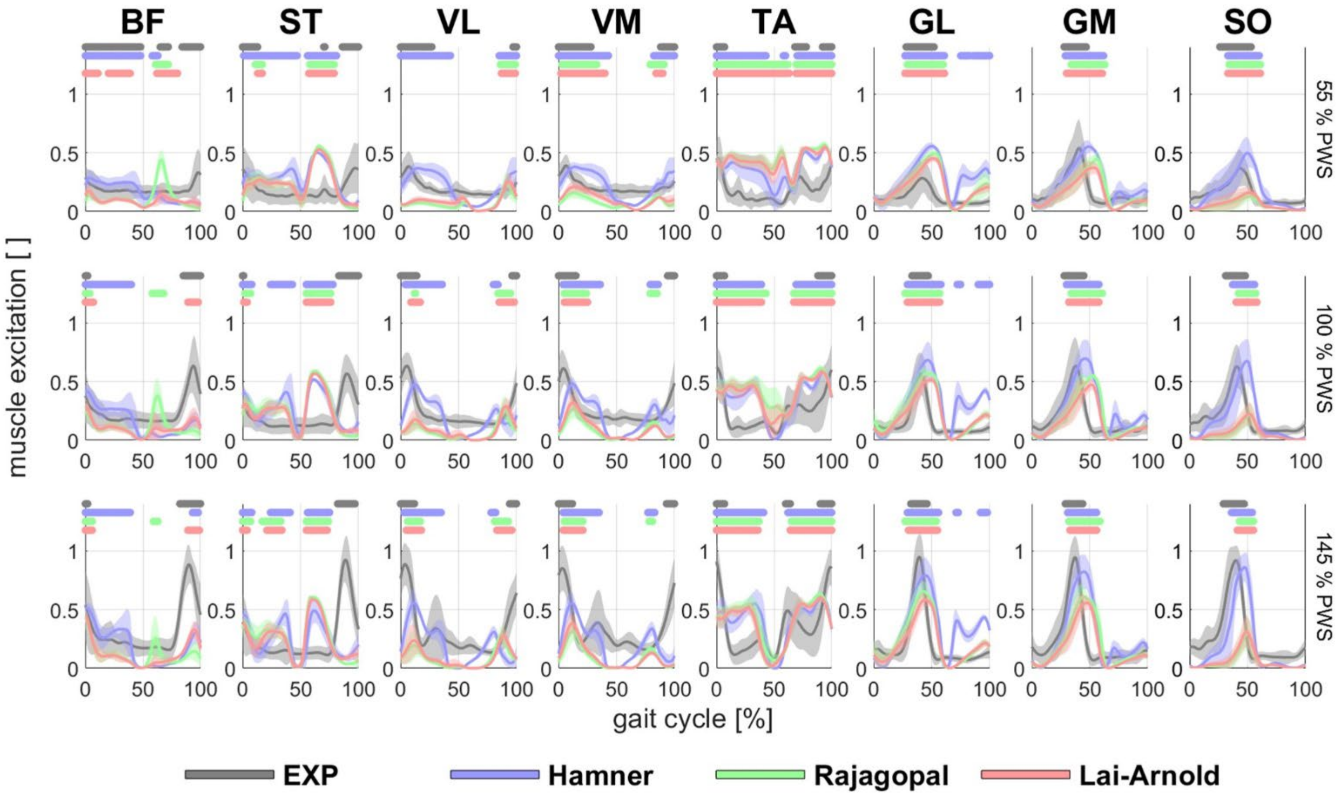

Supplementary Figure S1. EMGs and estimated/modelled muscle excitations (average +/- 1 SD of all subjects) in 8 muscles with performance criterion J10 and scaling variant S1 at walking speeds of 55%, 100% and 145% PWS. EMGs were normalized to the maximum value at 145% PWS. Horizontal lines above each time series indicate on/off timing for EMG and each model, defined as >50% excitation.

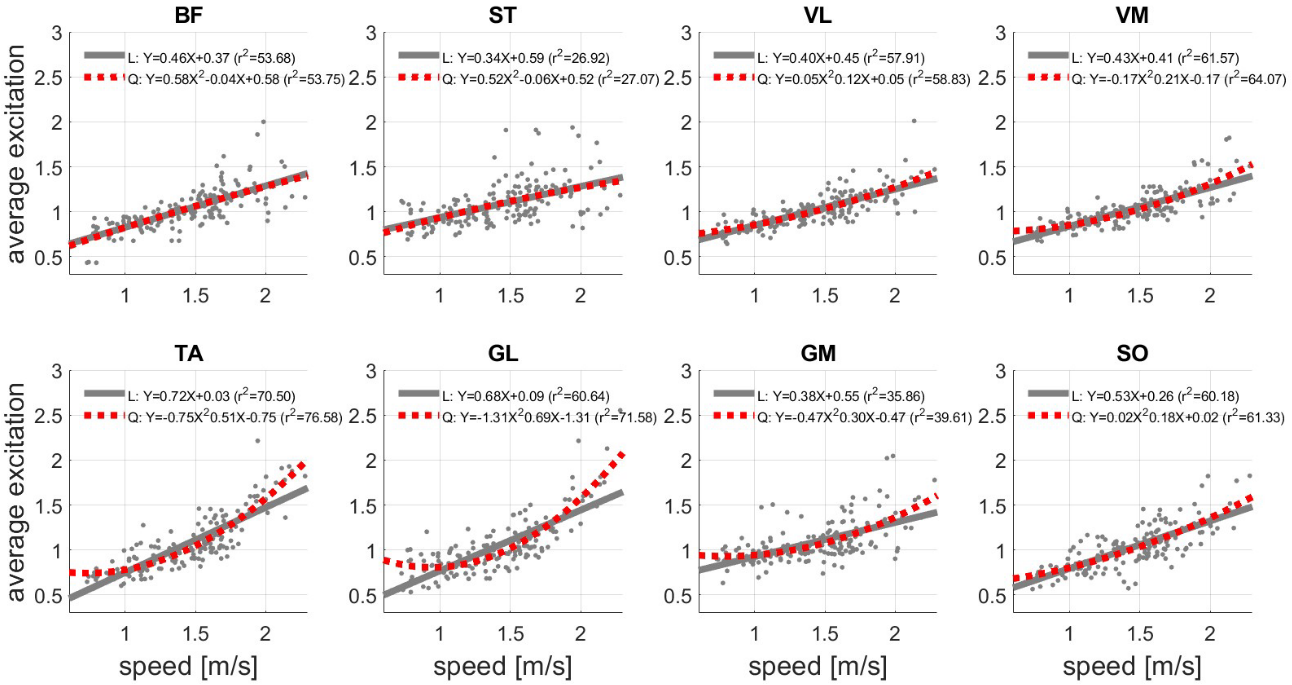

Supplementary Figure S2. Average EMG during the gait cycle versus walking speed for all subjects. Both linear and quadratic regressions are shown. Coefficients of determination with linear regression were similar to those with quadratic regression in 6 of 8 muscles, i.e., all except TA and GL. Linear regression was thus assumed to be sufficient to characterize trends of average muscle excitation over walking speeds. Normalization was performed based on the average value at PWS for each subject and muscle.

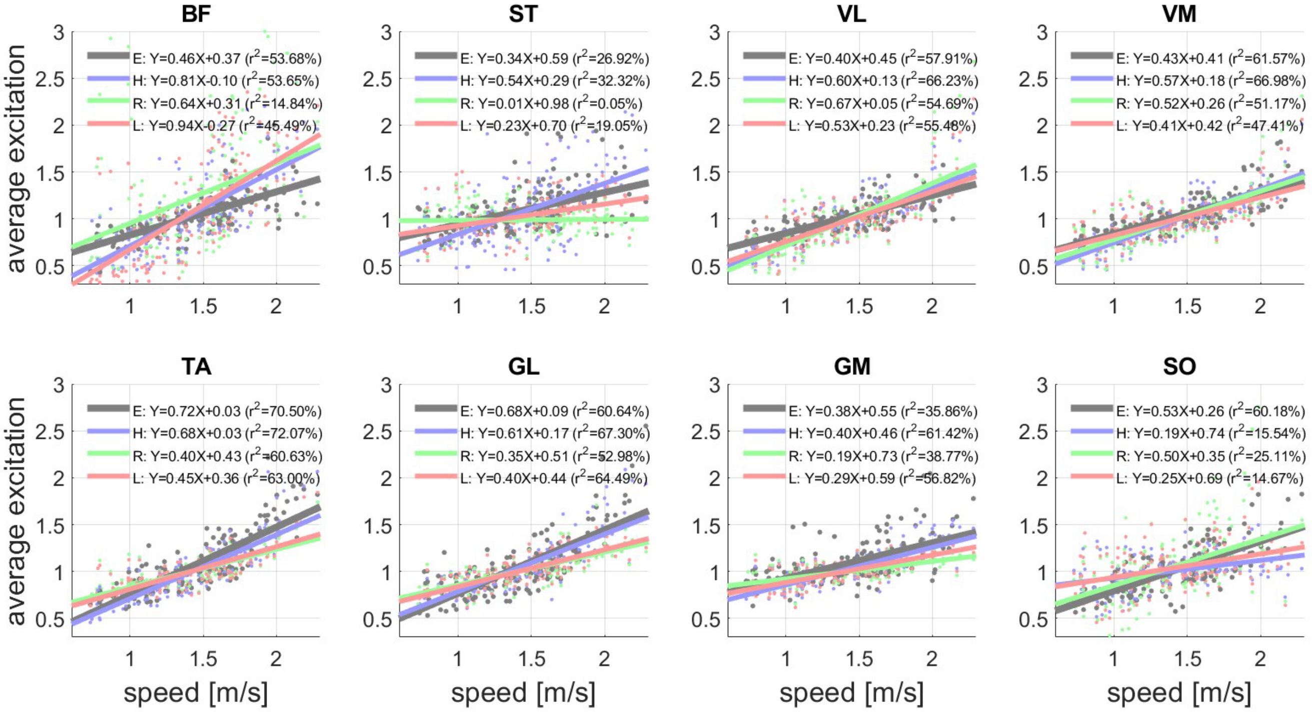

Supplementary Figure S3. Average EMGs and average estimated muscle excitations during the gait cycle versus walking speed for all subjects in 8 muscles with performance criterion J2 and scaling variant S1. Similar linear trends with respect to walking speed are found with other performance criteria and scaling variants. Average EMGs and average estimated muscle excitations were normalized to their values at 100% PWS.

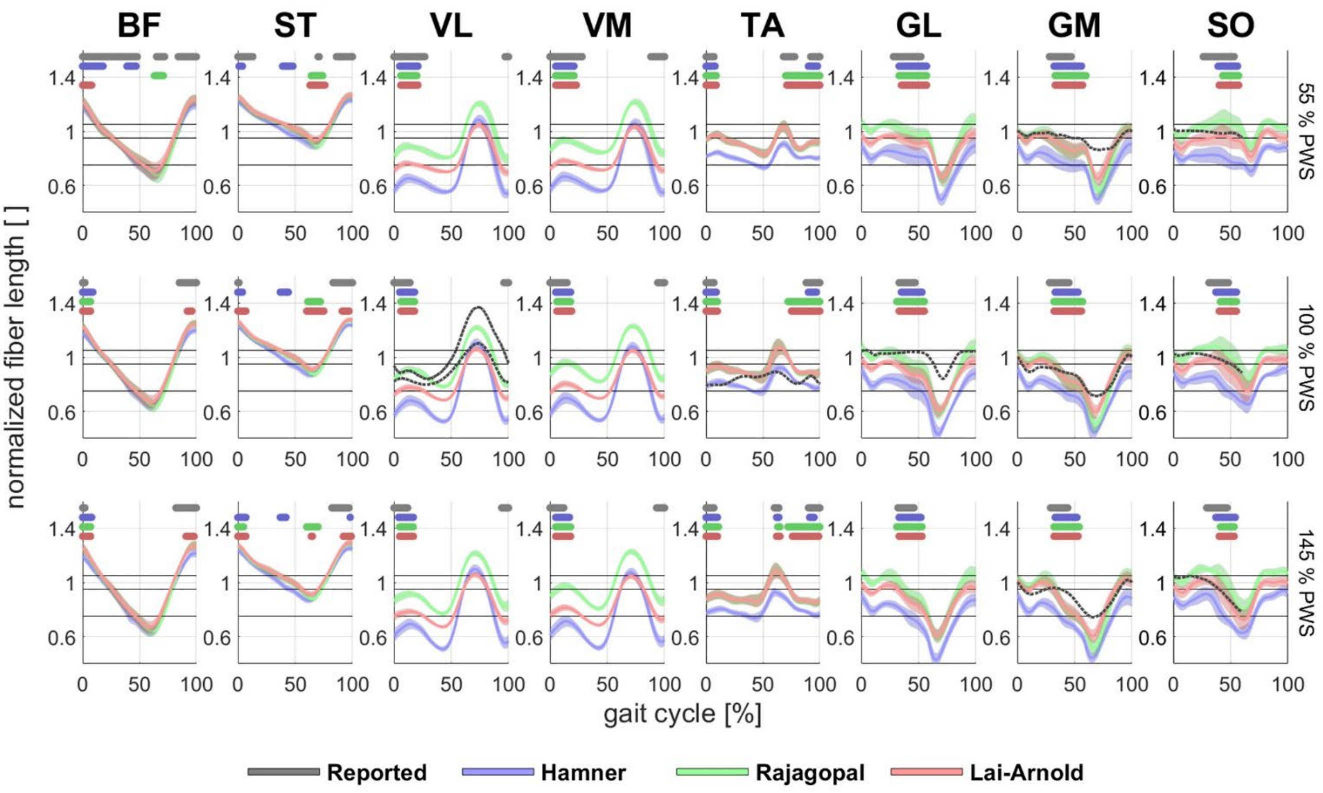

Supplementary Figure S4. Estimated normalized fiber length (average +/- 1 SD of all subjects) in 8 muscles with performance criterion J2 and scaling variant S2 at walking speeds of 55%, 100% and 145% PWS. Reported experimental normalized fiber lengths of SO (Lai et al., 2015), GM (Farris and Sawicki, 2012), GL (Farris and Raiteri, 2017), and VL (Chleboun et al., 2007; Bohm et al., 2018) are shown. Horizontal lines above time series indicate on/off timing for EMG and each model. Dashed horizontal lines indicate operating ranges between steep and shallow ascending limb (lower), shallow ascending limb and plateau (middle), and plateau and descending limb (upper).

References

Bohm, S., Marzilger, R., Mersmann, F., Santuz, A., and Arampatzis, A. (2018). Operating length and velocity of human vastus lateralis muscle during walking and running. *Sci. Rep.* 8, 1–10. doi: 10.1038/s41598-018-23376-5.

Chleboun, G. S., Busic, A. B., Graham, K. K., and Stuckey, H. A. (2007). Fascicle length change of the human tibialis anterior and vastus lateralis during walking. *J. Orthop. Sports Phys. Ther.* 37, 372–379. doi: 10.2519/jospt.2007.2440.

Farris, D. J., and Raiteri, B. J. (2017). Elastic ankle muscle-tendon interactions are adjusted to produce acceleration during walking in humans. *J. Exp. Biol.* 220, 4252–4260. doi: 10.1242/jeb.159749.

Farris, D. J., and Sawicki, G. S. (2012). Human medial gastrocnemius force-velocity behavior shifts with locomotion speed and gait. *Proc. Natl. Acad. Sci. U. S. A.* 109, 977–982. doi: 10.1073/pnas.1107972109.

Lai, A., Lichtwark, G. A., Schache, A. G., Lin, Y. C., Brown, N. A. T., and Pandy, M. G. (2015). In vivo behavior of the human soleus muscle with increasing walking and running speeds. *J. Appl. Physiol.* 118, 1266–1275. doi: 10.1152/japplphysiol.00128.2015.
